# Structural Models of Zebrafish (*Danio rerio*) NOD1 and NOD2 NACHT Domains Suggest Differential ATP Binding Orientations: Insights from Computational Modeling, Docking and Molecular Dynamics Simulations

**DOI:** 10.1371/journal.pone.0121415

**Published:** 2015-03-26

**Authors:** Jitendra Maharana, Bikash Ranjan Sahoo, Aritra Bej, Itishree Jena, Arunima Parida, Jyoti Ranjan Sahoo, Budheswar Dehury, Mahesh Chandra Patra, Sushma Rani Martha, Sucharita Balabantray, Sukanta Kumar Pradhan, Bijay Kumar Behera

**Affiliations:** 1 Department of Bioinformatics, Orissa University of Agriculture and Technology,Bhubaneswar, Odisha, India; 2 Biotechnology Laboratory, ICAR-Central Inland Fisheries Research Institute, Barrackpore, Kolkata, West Bengal, India; 3 Biomedical Informatics Centre, Regional Medical Research Institute (ICMR), Bhubaneswar, Odisha, India; Institut Pasteur Paris, FRANCE

## Abstract

Nucleotide-binding oligomerization domain-containing protein 1 (NOD1) and NOD2 are cytosolic pattern recognition receptors playing pivotal roles in innate immune signaling. NOD1 and NOD2 recognize bacterial peptidoglycan derivatives iE-DAP and MDP, respectively and undergoes conformational alternation and ATP-dependent self-oligomerization of NACHT domain followed by downstream signaling. Lack of structural adequacy of NACHT domain confines our understanding about the NOD-mediated signaling mechanism. Here, we predicted the structure of NACHT domain of both NOD1 and NOD2 from model organism zebrafish (*Danio rerio*) using computational methods. Our study highlighted the differential ATP binding modes in NOD1 and NOD2. In NOD1, γ-phosphate of ATP faced toward the central nucleotide binding cavity like NLRC4, whereas in NOD2 the cavity was occupied by adenine moiety. The conserved ‘Lysine’ at Walker A formed hydrogen bonds (H-bonds) and Aspartic acid (Walker B) formed electrostatic interaction with ATP. At Sensor 1, Arg328 of NOD1 exhibited an H-bond with ATP, whereas corresponding Arg404 of NOD2 did not. ‘Proline’ of GxP motif (Pro386 of NOD1 and Pro464 of NOD2) interacted with adenine moiety and His511 at Sensor 2 of NOD1 interacted with γ-phosphate group of ATP. In contrast, His579 of NOD2 interacted with the adenine moiety having a relatively inverted orientation. Our findings are well supplemented with the molecular interaction of ATP with NLRC4, and consistent with mutagenesis data reported for human, which indicates evolutionary shared NOD signaling mechanism. Together, this study provides novel insights into ATP binding mechanism, and highlights the differential ATP binding modes in zebrafish NOD1 and NOD2.

## Introduction

Innate immune system of higher organisms that restricts the proliferation of invading foreign pathogens usually senses pathogen associated molecular patterns (PAMPs) employing different pattern recognition receptors (PRRs). These molecular patterns are associated with pathogens or danger signals and conquer their space in the extracellular and cytoplasmic regions. The NOD-like receptors (NLRs), absent in melanoma (AIM)-2-like receptors (ALRs) and retinoic acid-inducible gene I (RIG-I) like receptors (RLRs) together constitute the intracellular PRRs. Whereas, extracellular PRRs comprises of Toll-like receptors (TLRs) and C-type lectin receptors (CLRs), which are widely distributed in the cell membrane and some TLRs (TLR3, 7, 8 and 9) are found in lysosomes and endosomes [1,2]. The NLR family members show a tripartite domain architecture with a C-terminal ligand binding domain (LBD) having variable numbers of leucine rich repeats (LRRs); a centrally positioned NACHT domain (found in NAIP, CIITA, HET-E and TP1), which oligomerizes in a nucleotide dependent fashion; and an N-terminally located effector binding domain (EBD) that accelerates the interaction with downstream effectors to initiate signaling cascade [3–5].

As in higher animals, the sub-cellular localization of PRRs plays a major role in the defense mechanism of lower vertebrates like fish. In fish, the defense system is empowered by various PRRs that recognize various PAMPs. Among the diverse members of PRR family, NLRs located in the cytoplasm play a major role in recognizing the invading PAMPs that intrude through the plasma membrane. NLRs trigger signaling processes ensuing innate immune responses upon interaction with the bacterial cell wall substructures [6–12]. The molecular mechanism of signaling is switched on only after the nucleotide binding and oligomerization of NACHT domain. The enhancement of NACHT oligomerization transfers the signal to EBD for further downstream signaling [13].

Several studies have indicated that NOD1 and NOD2 are well characterized PRRs, which play the most important roles in innate immune system [7,8]. It has also been revealed that certain mutations at amino acid level of NOD2 result harmful diseases like Chron’s disease (CD), Blau Syndrome (BS), and Early-onset sarcoidosis (EOS) [14–16]. Generally, before pathogenic invasion NOD proteins remain indolent with a folded LRR domain that is bound to NACHT domain. The innate immune signaling pathway starts when the disintegrated units of bacterial cell wall peptidoglycan (PGN), called iE-DAP and MDP, bind to the C-terminal concave surface of NOD1 and NOD2-LRRs [7–10]. Upon recognition of PGN, the LRR domain alters its conformation resulting in the exchange of ADP to ATP in NACHT domain. Further, ATP-bound NODs oligomerize to form an active platform that enhances the downstream signaling leading to NF-κB activation [17,18]. In a recent study, Zurek *et al*. has reported that NOD1 and NOD2-NACHT show different modes of activation upon ATP binding. Moreover, this group identified the critical residues responsible for nucleotide binding and also found the differential expression of NOD1 and NOD2 in response to ATP [19]. Even the activation of NOD1 and NOD2 ensues by direct interaction of bacterial peptidoglycans (iE-DAP and MDP) followed by nucleotide binding and oligomerization of NACHT and interaction of adaptor molecule (RIP2) with CARD domain; several proteins *viz*., heat shock protein (HSP90), SGT-1, SSH-1 etc. also involved in stability, autoinhibition, and various signal transduction processes [20–22]. Diverse aspects of ligand recognition, stability-instability of proteins, inhibition, autoinhibition, and enhancement of different signaling mechanisms modulated by NOD1 and NOD2 has been summarized in a recent review by Boyle *et al*. [23].

Despite NOD1 and NOD2 being the well-studied members of NLR family, several crucial aspects of protein-protein and protein-ligand interactions involving NOD proteins are still unclear. In order to study the *in vivo* relevance of NLR proteins the zebrafish model has been increasingly used [24,25]. Previously, we have predicted the interaction between bacterial PGNs (iE-DAP and MDP) with zebrafish NOD1 (zNOD1) and zNOD2-LRRs [26,27].

In the present study, we have investigated the molecular basis of ATP binding in zNOD1 and zNOD2-NACHT domains. The NACHT domains of zNOD1 and zNOD2 were modeled using comparative modeling approach. The binding modes between ATP and zNOD1 and zNOD2-NACHT domains were predicted using molecular docking followed by long-range molecular dynamics (MD) simulations. The findings of our computational study was supplemented with experimental findings of mouse NLRC4 (mNLRC4 [28]; a recently solvated structural homologue of zNOD1 and zNOD2) both in apo and holo conformation. We have hypothesized the differential mode of ATP binding, structural rearrangement, conformational entropy and participation of critical amino acids in zNOD1 and zNOD2-NACHT responsible for molecular recognition of ATP. To the best of our knowledge, this is the first ever report which provides mechanistic insights into the structural and molecular features of zNOD1 and zNOD2-NACHT domains. It is expected that our findings will enrich the present knowledge on tertiary architecture of NACHT domain and its ATP binding modes in a broader way.

## Material and Methods

### A. Sequence retrieval and domain search

The amino acid sequences of zNOD1 and zNOD2 were retrieved from NCBI protein database (GenBank ID: XP_002665106 and XP_697924). Domain prediction tools such as CD-search [29], SMART [30] and InterProScan [31] were used to identify the CARD(s), NACHT and LRR domains. Multiple sequence alignment of human, mouse and zebrafish NOD sequences were performed using MAFFT [32,33] and the critical residues and domain conservations were probed. Furthermore, the conservation of protein hot-spot residues involved in protein-ligand interactions and consistencies of ATP binding motifs in NACHT of NOD1 and NOD2 were compared with those reported in literatures [19,34].

### B. Structure prediction

The suitable templates for zNOD1 and zNOD2-NACHT model building were searched using BLASTp [35] program against PDB (http://www.pdb.org/) database. Due to low sequence identity between target and template (mNLRC4; PDB ID: 4KXF [28]), the protein sequences were submitted to automated model building servers like SWISS-MODEL [36], RaptorX [37] and I-TASSER [38]. The obtained 3D models were equated and evaluated based on discrete optimized protein energy (DOPE) scores. The zNOD1 and zNOD2-NACHT models with lowest DOPE value were considered for further structural refinement. GalaxyRefine [39] and WHATIF [40] programs were used for model refinement and side chain optimization. Further, secondary structures of zNOD1 and zNOD2-NACHT were predicted using PSIPRED [41] and compared with their respective 3D structure of template.

### C. Model validation

The energy-optimized models of zNOD1 and zNOD2-NACHT were verified for stereochemical quality using SAVeS (http://nihserver.mbi.ucla.edu/SAVES/), ProSA [42] and ProQ [43] web servers. The analysis of bond lengths and angles of the optimized-models were carried out in MolProbity [44]. In addition, the Z-score of H-bond energy, packing defect, bump score, radius of gyration (Rg) and deviation of Θ angles of the refined models were verified in VADAR [45], GeNMR [46] and PROSESS [47] web servers.

### D. Molecular docking

The two dimensional (2D) structure of ATP (CID 5957) was retrieved from PubChem database (https://pubchem.ncbi.nlm.nih.gov/) and 3D coordinates were constructed using OpenBabel v2.3.0 [48]. The modeled ATP was energy minimized and processed at PRODRG2 server to incorporate chirality and full charge [49]. Further, ATP was optimized using AutoDock 4.2 [50] and used for docking simulations. The docking parameters were acquired from earlier studies [26,27]. The information on ATP binding site was assumed from reported literatures [8,19,51]. The binding site was represented by three dimensional grid boxes. In addition to the automated dockings, manual dockings were also performed in reference to the experimental structure of ADP bound mNLRC4. Further, to validate our docking predictions, the ADP was replaced manually by ATP in crystal structure of mNLRC4-NACHT domain. In this approach, ATP molecule was placed carefully at the active site with charged atoms separated by ~3 Å and the intermolecular bumps were cleaned using DS Visualizer 3.5 (Accelrys Software, Inc.). From the automated docking calculations two each complexes from zNOD1 and zNOD2 were selected for MD simulation based on the free energy of binding, H-bonding and interatomic-bonding pattern, and a total of seven ATP bound complexes (six from both zNOD1/2, and one from mNLRC4) were selected for further optimization by employing long term MD simulation.

### E. Molecular dynamics simulations

MD simulations for zNOD1, zNOD2, mNLRC4-NACHT, and their ATP-bound complexes (*i*.*e*. 10 systems comprised of three apo and seven holo conformers) were performed using GROMACS 4.5.5 package [52] with Gromos96–43a1 force field. The topology of ATP was generated using PRODRG2 server. The simulation systems were solvated with SPC/E water model [53] in cubic boxes with minimum distance of 10 Å from the protein surface and box edge. A physiological strength (0.15 M) of counter ions was added to neutralize each simulation system. The atomic composition of the simulation systems is listed in S1 Table. The electro-neutralized simulation systems were subjected to steepest descent energy minimization to remove steric conflicts between atoms and to avoid high energy interactions until a tolerance of 1000 kJ/mol is reached. The energy minimized systems were then subjected to position restrained simulation in two different phases, NVT and NPT. NVT ensemble was used for temperature equilibration by restraining the positions of backbone atoms for 100 ps followed by pressure equilibration using NPT ensemble for 100 ps with backbone restraints applied. The production MD runs were performed for 50 ns keeping temperature of the systems 300 K *via*, Berendsen temperature coupling scheme, and the pressure of the systems was maintained at 1 bar using Parrinello-Rahman algorithm. All bond lengths were constrained using the LINCS algorithm [54], and SETTLE algorithm [55] was used to construct the geometry of water molecules. The Particles Mesh Ewald (PME) was used for electrostatic calculations and the simulations were run with periodic boundary conditions. The trajectory analysis was carried out using the built-modules of GROMACS and visual molecular dynamics (VMD 1.9.1) [56]. Grace 5.1.23 program (http://plasma-gate.weizmann.ac.il/Grace/) was employed for generation of 2D plots, whereas PyMOL 1.3 (www.pymol.org) and DS Visualizer 3.5 were used for visual inspection and interaction analysis.

### F. Conformational entropy

Conformational entropies of zNOD1, zNOD2 and mNLRC4-NACHT domains in both apo and holo states were calculated to probe the variation of structural stability in term of thermodynamic approximation using CENCALC v0.2.2 [57] software package. The calculation of conformational entropy was computed based on the changes in dihedral angles throughout the simulation time scale. This study considered the time dependent changes in two major dihedral angles, phi (φ) and psi (ψ) that basically control the rotations of the polypeptide backbone around the bonds between N-Cα and Cα-C, respectively for a proper biophysical estimation of apo and holo conformation.

### G. Essential dynamics

Essential dynamics (ED) or Principal component analysis (PCA) was performed to understand the global motion of the atomic coordinates of zNOD1, zNOD2 and mNLRC4-NACHT during MD simulation [58]. The main chain atoms of the protein molecules were considered for this analysis. The covariance matrix was constructed and diagonalized using *g_covar* and *g_anaeig* programs, respectively to generate the eigenvectors and eigenvalues. The prominent mobile regions of both proteins were carefully inspected and interpreted from the PCA and covariance matrix data.

## Results and Discussion

### A. Domain analysis

**Fig 1:**
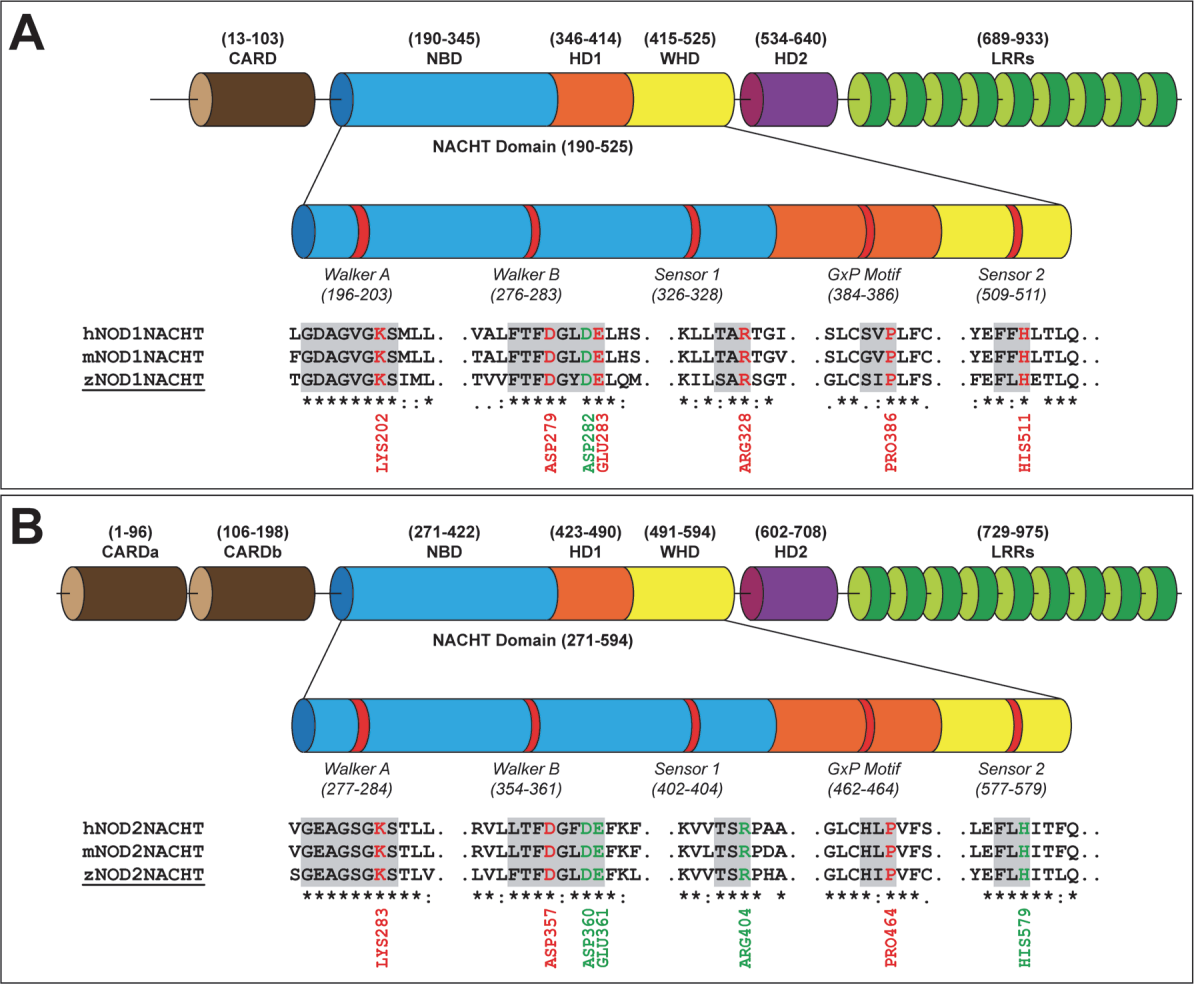
Domain architectures of zNOD1 (A) and zNOD2 (B). The NACHT domain is characterized by five different functional motifs. Multiple sequence alignment of zebrafish, human and mouse NACHT domains is constructed using MAFFT and the conserved functional motifs are highlighted. The highlighted residues represent the potential ATP binding sites and the conserved residues are shown as ‘*’. The ATP-binding residues conserved in all three species are labeled.

The protein sequences of zNOD1 and zNOD2 are composed of 940 and 980 amino acids, respectively. The sequence and domain comparison of zebrafish NODs with human and mouse revealed tripartite domain architectures, *i*.*e*. N-terminal CARD/s, middle NACHT and C-terminal LRRs (S1 Fig.). NOD1 has one CARD domain (13–106), whereas NOD2 contains two; CARDa (1–96) and CARDb (106–195). The NACHT domain of both receptors consists of three sub-domains; N-terminal nucleotide-binding domain (NBD), middle helical domain 1 (HD1) and C-terminal winged helix domain (WHD) well connected by highly variable linkers. The NACHT domain and extended HD2 forms the NOD, which is indispensable for oligomerization activity. The sequence identities and similarities of critical nucleotide binding motifs, Walker A, Walker B, Sensor 1, GxP motif and Sensor 2 are depicted in Fig. 1. Multiple sequence alignment presented substantial residue conservation at Walker A box (GxxxxGK[S/T]; where x is any amino acid), which was reported to be critically engaged in ATP binding [59]. Moreover, the so called ATP interacting sites (in NLR proteins) *i*.*e*. Walker B (hhhhD/E; where, ‘h’ denotes a hydrophobic residue) or, extended Walker B box (DGhDE), Sensor 1, GxP motif and Sensor 2 were also found to be fairly conserved [13,19,60]. The key ATP binding charged and polar residues in human NOD1 (hNOD1) and hNOD2-NACHT were well conserved in zebrafish, indicating a conserved nucleotide binding pocket in both zNOD1 and zNOD2.

### B. Model construction and validation

The crystal structure of mNLRC4 (PDB ID: 4KXF) [28] was found to be the best template for modeling the NACHT domain. The built models with lowest DOPE scores (S2 Fig.) were evaluated as good model that were energy optimized in GalaxyRefine server. The missing side chains were modeled in WHATIF. The secondary structure components as predicted by PSIPRED (S3 Fig.) presented a good structural correlation with the constructed 3D models. The model validation scores of refined models indicated that the stereo-chemical parameters were reasonably good. The backbone dihedral angle analysis of zNOD1 and zNOD2-NACHT models was performed using Ramachandran plot [61] of PROCHECK [62]. The results revealed that, zNOD1 and zNOD2-NACHT models showed 97.4 and 97.6% of residues within the allowed regions, respectively (Table 1, S4 Fig.). Verify3D scores of these models exhibited a good agreement to their primary sequences. ERRAT analysis provided a good non-bonded interaction statistics for both models. The Z-score reports from ProSA indicated that the models were within the acceptable range of X-ray and NMR. The LGscore and MaxSub from ProQ analysis indicated a good model quality. MolProbity server indicated no bad bond lengths and angles for the predicted models. The H-bond energy, packing defect, bump score, radius of gyration (Rg) and deviation of Θ angles calculated at VADAR, GeNMR and PROSESS servers were within the cut-off range, as summarized in Table 1.

**Table 1:** Model validation scores of zNOD1-NACHT and zNOD2-NACHT of zebrafish.

**Servers**		**zNOD1-NACHT**	**zNOD2-NACHT**
PROCHECK	Most favored regions (%)	86.40	84.30
	Additionally allowed Regions (%)	9.90	9.40
	Generously allowed Regions (%)	1.00	3.80
	Disallowed regions (%)	2.60	2.40
	Overall G-factor	0.18	0.13
Verify3D	Averaged 3D-1D Score > 0.2	82.79	88.92
ERRAT	Overall Quality	84.45	89.87
ProSA	Z-Score	−7.57	−6.26
ProQ	LG score	3.16	4.63
	MaxSub	0.22	0.45
MolProbity	Cβ deviations >0.25Å (%)	0.31	1.32
	Residues with bad bonds (%)	0.00	0.00
	Residues with bad angles (%)	0.00	0.06
RESPROX	Predicted resolution(Å)	2.24	2.29
VADAR	Standard deviation of χ1 pooled	0.64	1.02
(Z-score)	Mean H-bond energy	0.65	0.65
	Generously allowed Ω angles (%)	0.52	−0.26
	Packing defects (%)	1.21	0.95
	Percentage of 95% buried residues	0.54	0.24
GeNMR	Ramachandran outside of most favored	1.81	2.67
(Z-score)	Bump score	0.34	0.34
	Radius gyration score	1.33	1.40
PROSESS	Deviation of Θ angles	0.86	1.07
(Z-score)	χ1 score	0.42	0.12

### C. Structural description

**Fig 2:**
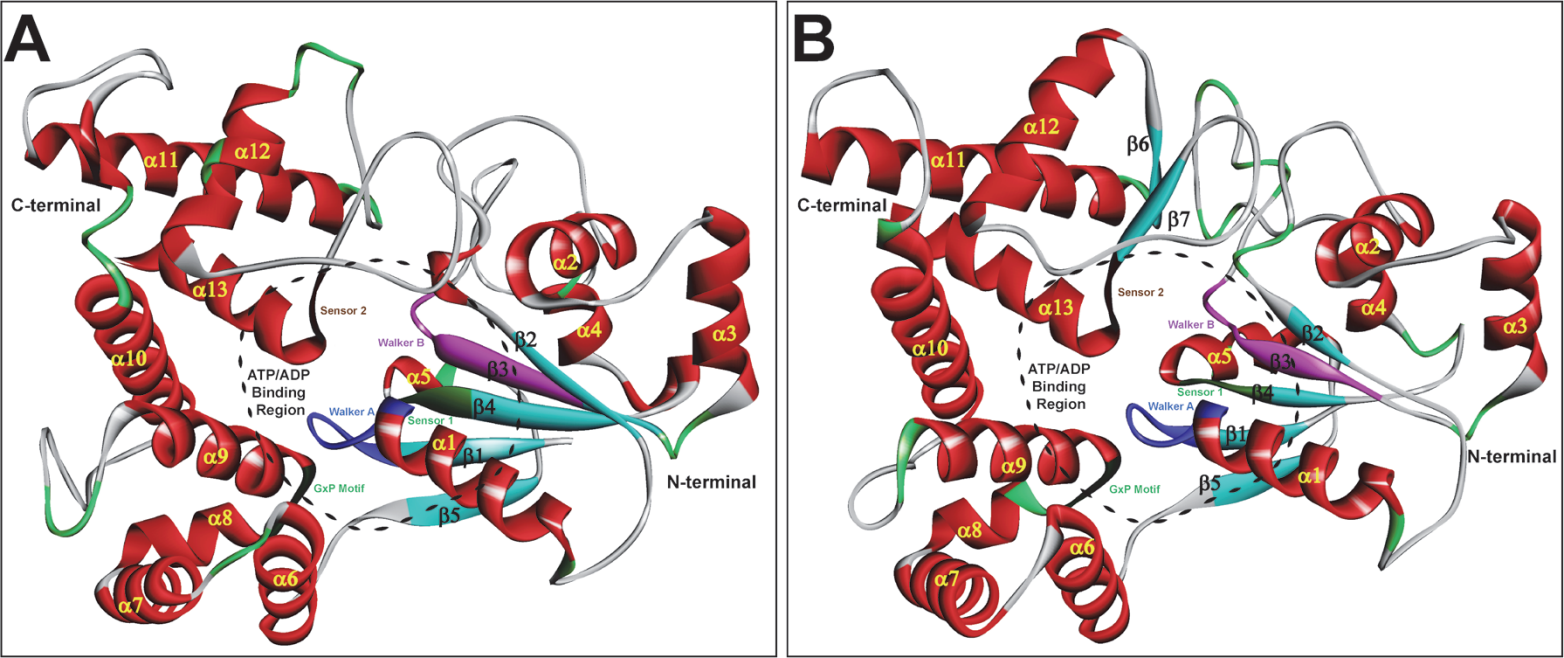
The 3D models of zNOD1-NACHT (A) and zNOD2-NACHT (B) domain. The protein is shown as solid ribbon with α-helices (in red), β-sheets (in cyan), and turns (in white). The five different functional motifs are highlighted with different colors and are labeled. Walker A is shown in blue, Walker B in magenta, Sensor 1 in green, GxP motif in deep green, and Sensor 2 in deep blue. The ATP binding pocket is displayed with dashed line.

The validated models of zNOD1 and zNOD2-NACHT domains exhibited approximately same spatial arrangements along-with conserved structural units. The overall architecture of both NACHT domains can be structurally categorized into three discrete sub-domains *viz*., N-terminal NBD, middle HD1 and C-terminal WHD (Fig. 2). The N-terminal domain of both models is comprised of five parallel β-sheets (β1002Dβ5) and four α-helices (α1-α4), and two additional 3_10_ helices were noticed in NOD1-NACHT. In both NOD1 and NOD2-NACHT models, each of the helical domains *i*.*e*., HD1 and WHD were comprised of four α-helices extending from α6-α9, and α10-α13 respectively. The C-terminal β-sheets (β6-β7) in NOD2-NACHT were found to be absent in NOD1. The functional motifs, Walker A, Walker B and Sensor 1 were located in NBD, whereas GxP motif was in HD1 and Sensor 2 was positioned in WHD (Fig. 1, 2).

### D. Analysis of docking results

**Fig 3:**
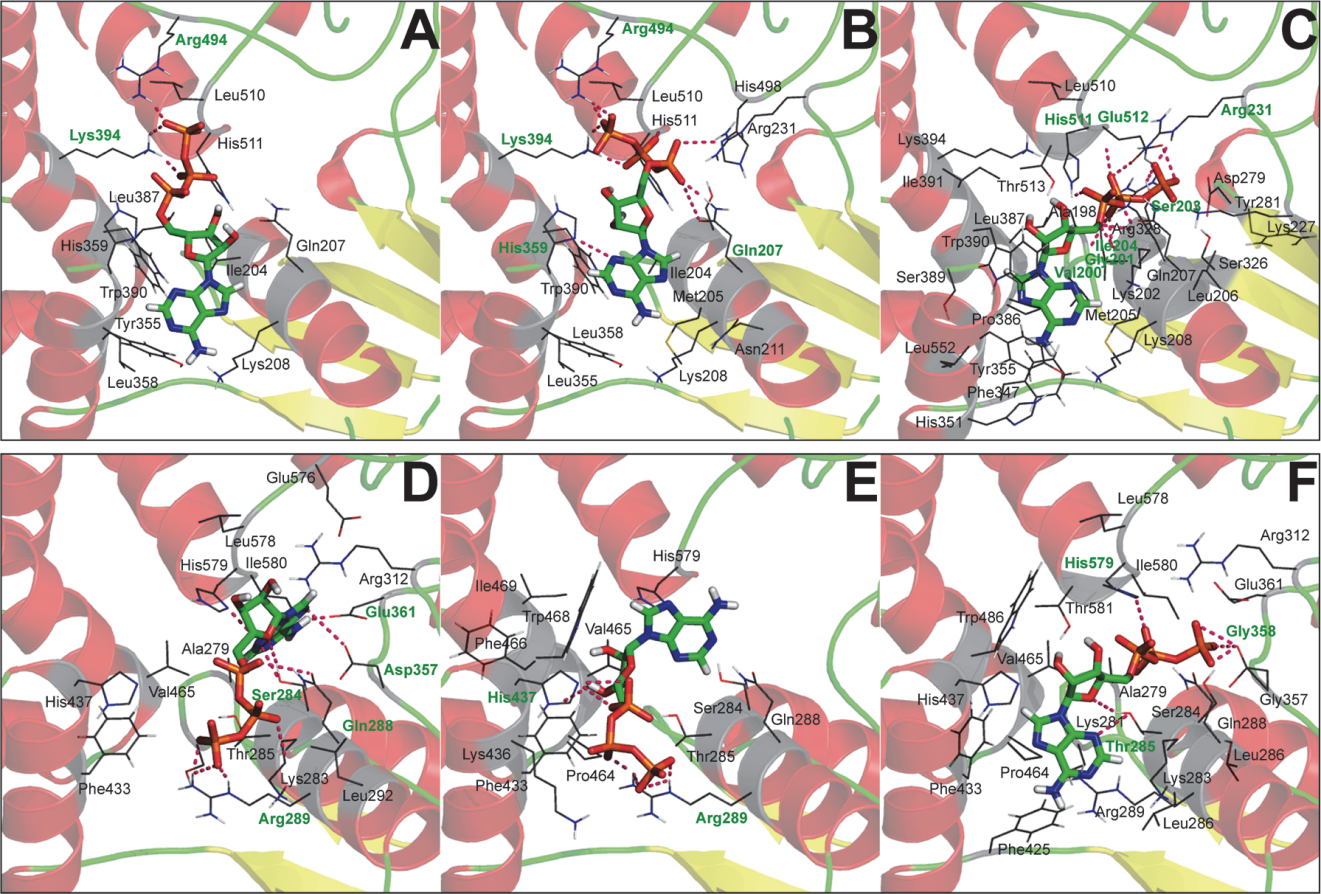
Molecular interaction of ATP with zNOD1 and zNOD2-NACHT domains. (A) Complex I for NOD1-NACHT, (B) Complex II for NOD1-NACHT, (C) Complex III for NOD1-NACHT, (D) Complex I for NOD2-NACHT, (E) Complex II for NOD1-NACHT, and (F) Complex III for NOD1-NACHT. The graphics is generated using PyMOL. The ATP molecule is shown as stick, protein as cartoon, and H-bonds as red dotted lines. The amino acids marked in green are designated as H-bond forming residues.

Docking simulation of ATP with NOD1 and NOD2-NACHT provided promising binding affinity at conserved ATP binding sites, which is an agreement with the recent studies by Zurek *et al*. and Mo *et al*. [8,19]. The docking scores, interacting amino acids, and binding modes of ATP have been displayed in Tables 2, 3, and Fig. 3. In zNOD1-NACHT, γ-phosphate group of ATP was deeply buried inside the central cavity, whereas adenine moiety was positioned outward from the core cavity (Fig. 3A, B). In contrast, ATP in zNOD2-NACHT showed reverse binding mode where the adenine moiety protrudes toward the binding cavity and γ-phosphate group extends away from the inner cavity (Fig. 3D, E). Mutational studies in ATP binding sites presented contrasting effects on NOD1 and NOD2 signaling and NF-κB activation [19]. Further, the group has also stated the mode of NF-κB signaling and binding mode of ATP is different in both NOD1 and NOD2 [19]. The auto activation of NOD2 signaling upon mutation of extended Walker B was shown to have no effect on NOD1 signaling. This suggested a differential ATP binding mode in potential Walker box regions of NOD1 and NOD2-NACHT [19]. Reported co-crystallized structures of mNLRC4 (close structural homologue of NOD1 and NOD2) revealed that β-phosphate group of ADP acquires the central cavity and adenine moiety poses outward from the centre [28]. To validate the predicted docking calculation by AutoDock, a manual docking simulation has also been performed by extrapolating experimental structure of ADP-bound mNLRC4 (Fig. 3C, F). So as to support the findings from molecular docking of zNOD1/2 with ATP, mNLRC4-ATP docked complex was considered for further optimization through MD simulation. A total of seven complexes including three of each NOD1 and NOD2-NACHT, and one from mNLRC4-NACHT-ATP were considered for further studies.

**Table 2:** Molecular docking results of ATP with zNOD1 and zNOD2-NACHT at different grid boxes using AutoDock.

**Solutions**	**Grid centre**	**Binding Energy**	**Ligand efficiency**	**No. of H-Bond**	**H-bond forming residues**
zNOD1-NACHT-ATP					
1	35x40x45	−5.44	−0.18	3	Lys394
2	40x45x50	−5.98	−0.19	4	Lys394, Arg494
3	45x50x55	−6.18	−0.20	6	Gln207, His359, Lys394, Arg494
zNOD2-NACHT-ATP					
1	35x40x45	−4.09	−0.13	7	Ser284, Arg289, Glu361, His437
2	40x45x50	−5.86	−0.19	5	Gln288, Arg289, His437
3	45x50x55	−6.57	−0.21	6	Arg289, Lys436, His437

**Table 3:** Interaction analysis (hydrogen bond, hydrophobic contacts, electrostatics and van der Waals contact) of NOD1-NACHT-ATP and NOD2-NACHT-ATP complexes before molecular dynamics simulation.

**Complexes**	**Hydrogen bonds**		**Hydrophobic**	**Electrostatic**	**van der**
	**(H-bonds)**		**Interaction**	**Interaction**	**Waals Contacts**
	**NACHT**	**ATP**			
**zNOD1-NACHT-ATP Interaction**					
Complex I	Lys394:HZ1	O2	Leu510	Gln207,	Ile204,
	Lys394:HZ1	O6		His359,	Lys208,
	Lys394:HZ3	O3		His511	Tyr355,
	Arg494:HH21	O3			Leu387,
					Trp390
Complex II	Gln207:HE21	O7	Ile204,	Arg231,	Lys208,
	His359:HD1	N5	Leu510	His498,	Trp390
	Lys394:HZ1	O4		His511	
	Lys394:HZ2	O1			
	Arg494:HH21	O1			
	Arg494:HH21	O2			
Complex III	Lys202:HN	O5	Val200,	Lys208,	Ala198,
(Manual docking)	Ser203:HN	O9	Ile204,	Asp279,	Gly201,
	Ser203:HG	O2	Met205,	Ser326,	Leu206,
	Arg231:HE	O2	Tyr355,	His351,	Lys227,
	Arg231:HH21	O2	Leu387,	Pro386,	Gly280,
	Arg231:HH21	O2	Trp390	Lys394,	Arg328,
	His511:HE	O8		Thr513	Phe347,
	Glu512:HN	O4			Leu387,
					Ser389,
					Ile391,
					Leu510
**zNOD2-NACHT-ATP Interaction**					
Complex I	Ser284:HG	N5	Asp358,	Thr285,	Ala279,
	Arg289:HE	O3	Leu578,	Gln288,	Leu292,
	Arg289:HH22	O1	Ile580	Arg312,	Gly358,
	Arg289:HH22	N5		Asp357	Phe433,
	Glu361:OE1	H11			His437,
	Glu361:OE1	H10			Val465,
	His579:HD	N5			Glu576
Complex II	Gln288:HE22	N4	Phe433,	Ser284,	Phe466,
	Arg289:HE	O3	Val465,	Thr285,	Ile469
	Arg289:HH21	O4	Trp468	Lys436,	
	Arg289:HH21	O1		Pro464,	
	His437:HE2	O7		His579	
Complex III	Ser284:HG	O1	Ala279,	Ser281,	Gly280,
(Manual docking)	Thr285:HG1	N5	Asp357,	Lys283,	Gly282,
	Thr285:HG1	O11	Phe425,	Gln288,	Leu286,
	His579:HD1	O4	Phe433,	Arg289,	Arg312,
			Val465,	Asp357,	Gly358,
			Trp468,	Glu361,	Thr402,
			Ile580	His437,	Leu578,
				Pro464	Thr581

### E. Trajectory analysis

**Fig 4:**
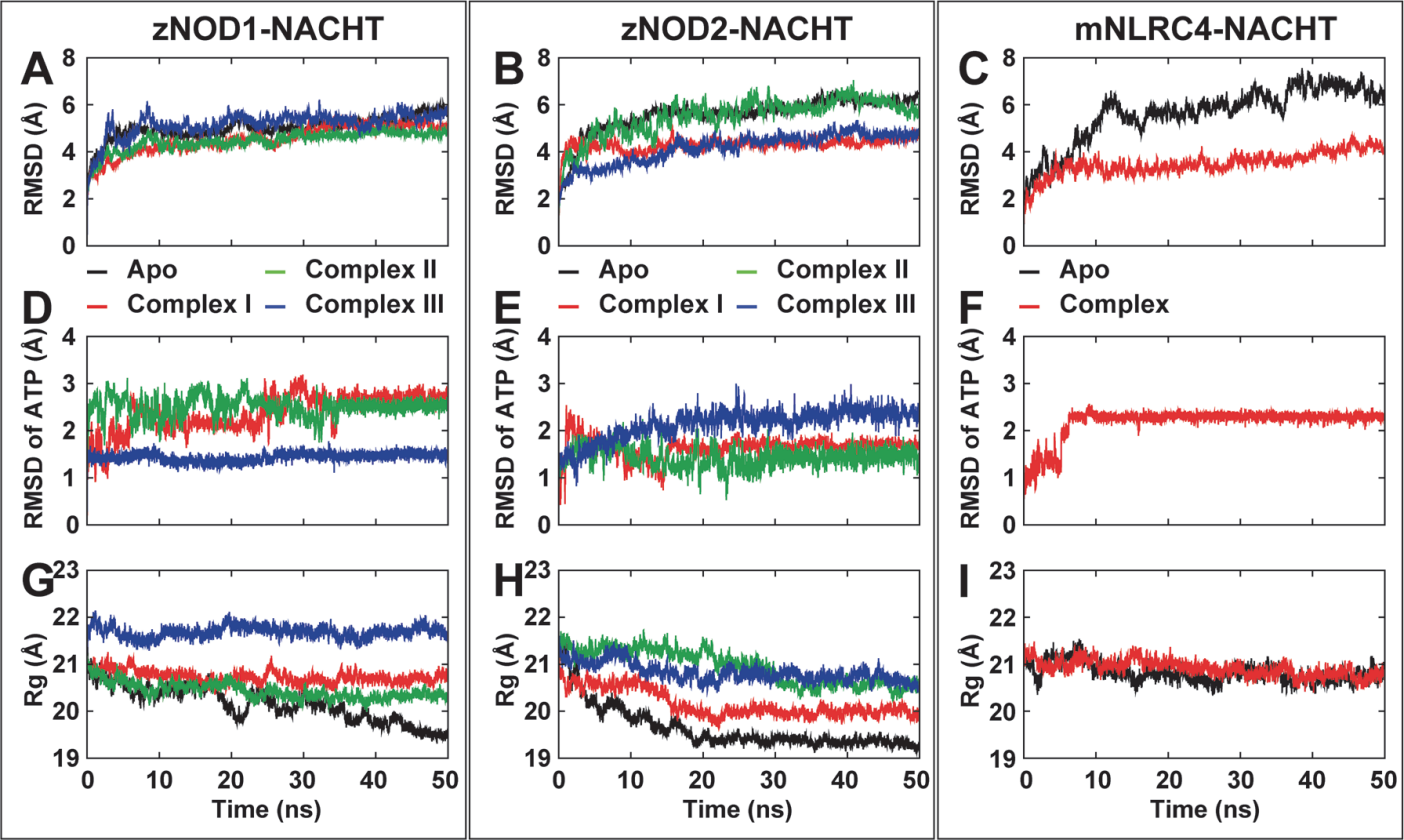
Conformational stability of NACHT-ATP complex throughout 50 ns time period. (A) Backbone-RMSD of zNOD1-NACHT, (B) Backbone-RMSD of zNOD2-NACHT, (C) Backbone-RMSD of mNLRC4-NACHT, (D) RMSD of ATP atoms in zNOD1-NACHT complex, (E) RMSD of ATP atoms in zNOD2-NACHT complex, (F) RMSD of ATP atoms in mNLRC4-NACHT complex (G) Radius of gyration (Rg) of zNOD1-NACHT, (H) Radius of gyration (Rg) of zNOD2-NACHT,and (F) Rg of mNLRC4-NACHT. All graphs are generated using Grace 5.1.23 plotting program.

The structural integrity and mechanical stability of zNOD1, zNOD2 and mNLRC4-NACHT in apo and holo states were thoroughly inspected to explore ATP-dependent self-oligomerization from their MD trajectories. In NOD1, RMSD of the apo system exhibited a constant backbone deviation of ~4 Å (as compared to the initial conformation) between 10 and 42 ns. Thereafter, the deviation slightly increased up to 50 ns with an average RMSD of ~4.5 Å. Among three ATP-bound NOD1 complexes, Complex I and II showed approximately similar backbone deviation pattern, but in Complex III (manual docking), the RMSD was found to be stable after 20 ns (Fig. 4A). Unlike NOD1, the NOD2 apo system showed comparatively higher RMSD for backbone atoms (deviated up to ~5.2 Å). Among three holo systems, Complex I presented a stable backbone conformation after 15 ns with a lowest RMSD value in comparison to Complex II and III (Fig. 4B). As compared to the RMSD of zNOD1 and zNOD2, mNLRC4-NACHT showed a higher and unstable RMSD in apo (diverged up to ~7.4 Å), however in ATP-bound state, RMSD was found to be more stable (Fig. 4C). In addition to the backbone RMSD of proteins, the ligand-RMSD analysis was also performed to understand the stability of the ligand in the active site pocket. Among the NOD1-complexes, the Complex III showed highest stability throughout the simulation with a deviation of ~1.3 Å (Fig. 4D), whereas, RMSD of others became stable after 35 ns with a maximum deviation of ~2.7 Å. On the other hand, in NOD2-complexes, the RMSD of ATP was found to be unstable throughout the simulation. The Complex I exhibited a constant RMSD of ~1.5 Å after 15 ns; though Complex III was stable with the backbone deviation was found to be larger (~2.3 Å) as compared to Complex I and II (Fig. 4E). A constant RMSD of ATP was observed in mNLRC4 (~2.3 Å) just after 5 ns of MD simulation till 50 ns (Fig. 4F). In both the apo systems (of zNOD1 and zNOD2), the protein depicted a decreasing order of gyration radii, which suggested their shape and size were becoming compact (Fig. 4F, G). In NOD1 holo complexes, Complex III demonstrated a higher gyradius (~21.7 Å) as compared to rest two complexes. But, the NOD2-holo conformations demonstrated a small Rg value (~20.1 Å) for the Complex I with a stable gyradius after 15 ns. Unlike to NOD1 and NOD2, the gyradius of mNLRC4 portrayed a constant trend (more compact) in both apo and holo condition (Fig. 4I) indicating differential structural behaviors in dynamic condition in response ATP binding.

**Fig 5:**
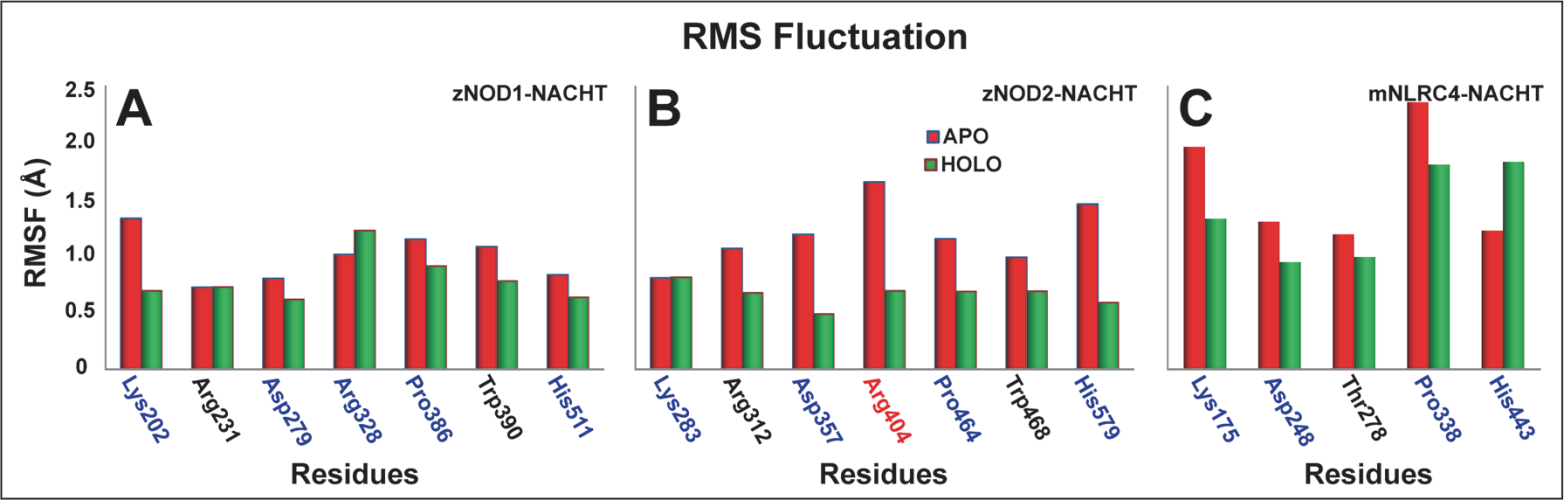
RMSF of critical residues involved in ATP binding. zNOD1-NACHT (A), zNOD2-NACHT (B) and mNLRC4-NACHT (C). The apo and holo conformations are shown as red and green respectively. The amino acids marked in blue are critical residues participate in ATP binding supported by experimental evidences, the residues marked in black may participate in ATP recognition have been proposed in this study and residues (Arg404 in zNOD2) didn’t form any interaction as compared to zNOD1.

**Fig 6:**
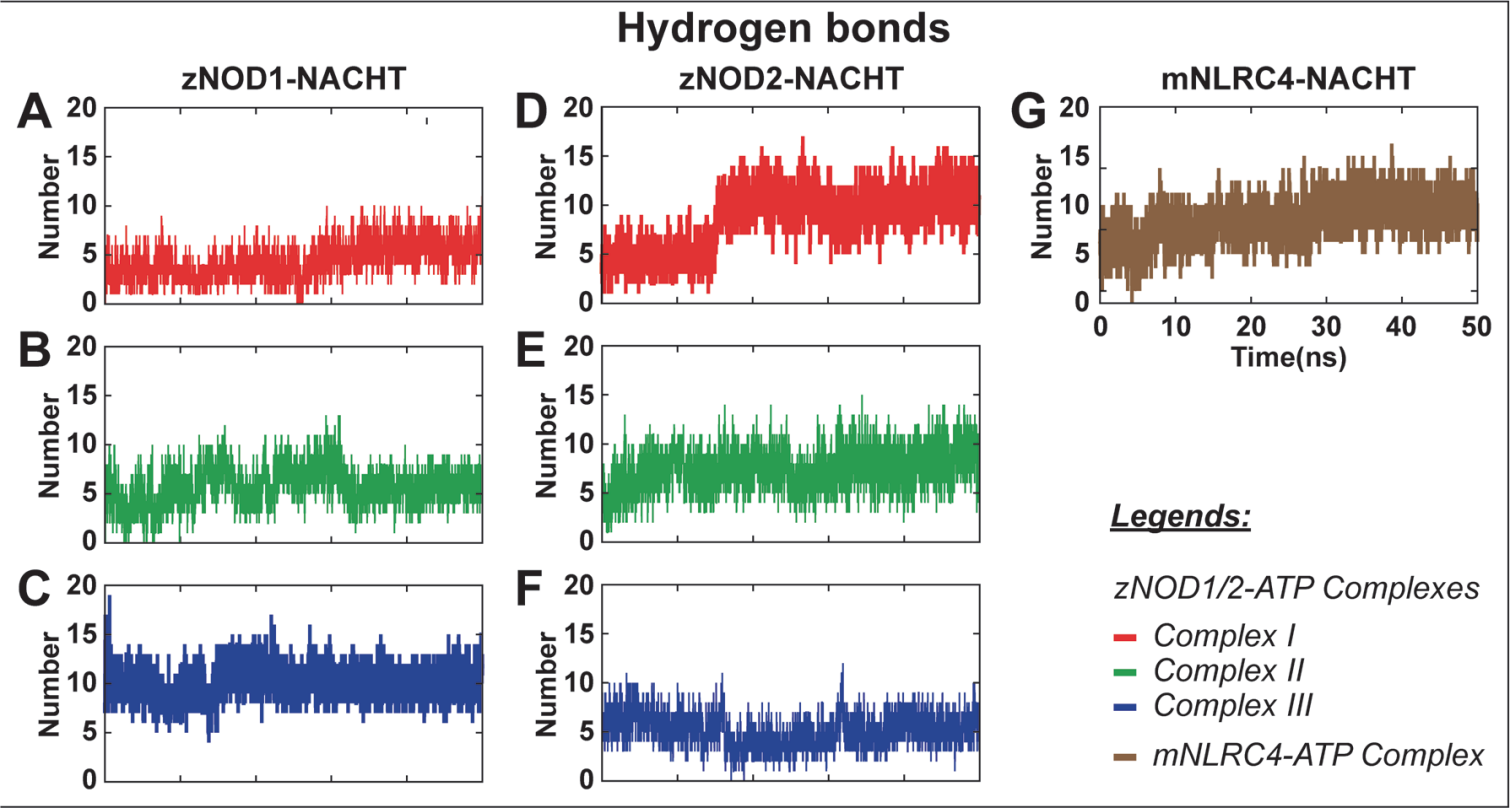
Variation of H-bonds participated in interaction during 50 ns simulation in zNOD-NACHT complexes. (A) Complex I for zNOD1-NACHT, (B) Complex II for zNOD1-NACHT, (C) Complex III for zNOD1-NACHT, (D) Complex I for zNOD2-NACHT, (E) Complex II for zNOD2-NACHT and (F) Complex III for zNOD2-NACHT (G) mNLRC4-NACHT complex. The y-axis represents the number of H-bond formed during the course of MD simulation and the simulation time (in ns) is depicted in x-axis. All graphs are generates using Grace 5.1.23 plotting program.

The Cα RMSFs were minutely analyzed for both apo and holo complexes of NOD1 and NOD2-NACHT domains from the 50 ns trajectories. In case of NOD1 (S5A Fig.), Complex III showed a comparatively lower fluctuation than that of apo and other complexes; whereas in NOD2 (S5B Fig.), Complex I displayed less Cα fluctuation than other two complexes. In both the complexes, (Complex III; NOD1 and Complex I; NOD2), the RMSFs of probable ATP binding regions showed minute fluctuations than the other complexes, signifying strong anchoring of ATP in the binding pockets during MD simulation. Like NOD1 (Complex III) and NOD2 (Complex I), we also found lower overall Cα RMSF in mNLRC4-ATP bound complex (S5C Fig.). In contrast to the apo form, critical assessment of the key ATP binding residues in these complexes, (*i*.*e*., Complex III of NOD1, Complex I of NOD2, and NLRC4-ATP complex) showed minimal fluctuation (Fig. 5A-C) with few exceptions. For instance, Arg404 of NOD2 Sensor 1 (Fig. 5B), which corresponds to Arg328 of NOD1 Sensor 1 (Fig. 5A) (responsible for sensing γ-phosphate of ATP) showed much more fluctuation in apo form as compared to the holo form. Due to absence of ‘Arg’ in ‘279’ position (in mNLRC4 Sensor 1), no interaction has been observed with ATP (as discussed below); but, adjacent Thr278 formed a strong electrostatic contact with ATP with lower RMSF in holo condition as compared to apo form. Furthermore, Sensor 2 ‘His’ (in zNOD1 and zNOD2) showed lower fluctuation in holo form as compared to apo form. However, in case of mNLRC4, conserved ‘His’ (His443) showed higher fluctuation in ATP bound condition (Fig. 6C), which seems to corroborate with the earlier findings of Hu *et al*. [28]. The secondary structure assessment of zNOD1 and zNOD2-NACHT domain in both apo and holo state were found to be strongly conserved during the course of MD simulations as evidenced from S6 Fig., reflecting the stability of secondary structural elements of the model in aqueous environment.

### F. Hydrogen bond (H-bond) analysis

Intermolecular H-bonds are the measure of binding stability between a protein and its binding components. To scrutinize the best holo conformation for both NOD1 and NOD2, we computed the consistency of H-bonding forces from MD trajectories. The H-bonds between NACHT and ATP in all complexes are illustrated in Fig. 6. In NOD1, the Complex III (Fig. 6C) displayed a greater number of H-bonds (average of ~10 numbers) as compared to Complex I and II (Fig. 6A, B). On the other hand, Complex I of NOD2 depicted higher number of H-bonds (average of ~8 numbers during 50 ns and ~10 in between 15–50 ns), as evident from long term MD simulation (Fig. 6D). As compared to zNOD1 and zNOD2 H-bond graph, mNLRC4-ATP docked complex also displayed an average of ~10 numbers of H-bonds (Fig. 6G) indicating their active participation in ATP recognition. Collectively, the H-bond analysis suggested better binding mode between NACHT and ATP in Complex III of NOD1 and Complex I of NOD2, which were considered for further analysis.

### G. Conformational entropy calculation

The conformational entropy was computed for both zNOD1 and zNOD2-NACHT in apo and holo state to get a better approximation of thermodynamic favorable conformers. The results suggested that the conformational entropy of Complex III in NOD1 and Complex I in NOD2 showed minimum level of entropy (against apo conformations) (Table 4), which is strongly correlated with the RMSD, RMSF, Rg and H-bond analysis. Besides that, it was also found that in zNOD1 and zNOD2 the proper ATP-bound conformation showed a plenty of reduction in entropy (32.5% in zNOD1, 10.2% in zNOD2 NACHT) as compared to its apo conformation. Further, the conformational entropy of NOD2 was noticed to be higher than that of zNOD1 in both apo and holo conditions; which suggests a differential role of NOD1 and NOD2 in ATP dependent structural modulation of NACHT/NOD domain to initiate self oligomerization and NOD signaling.

**Table 4:** Conformational entropy of zNOD1 and zNOD2-NACHT domain under apo and holo conditions. Bold fonts depicts minimum level of entropy.

**Conformations**	**Conformational Entropy (cal/mol K)**		
	**Phi (φ)**	**Psi (ψ)**	**Average**
zNOD1-NACHT			
apo	50.81	59.24	55.03
Complex I	33.46	52.43	42.95
Complex II	51.82	50.08	50.95
**Complex III**	**38.04**	**36.25**	**37.15**
zNOD2-NACHT			
apo	69.88	87.25	78.57
**Complex I**	**64.21**	**76.88**	**70.55**
Complex II	77.29	96.79	87.04
Complex III	72.42	78.31	75.37

### H. Interaction analysis of zNOD1 and zNOD2-NACHT with ATP

**Fig 7:**
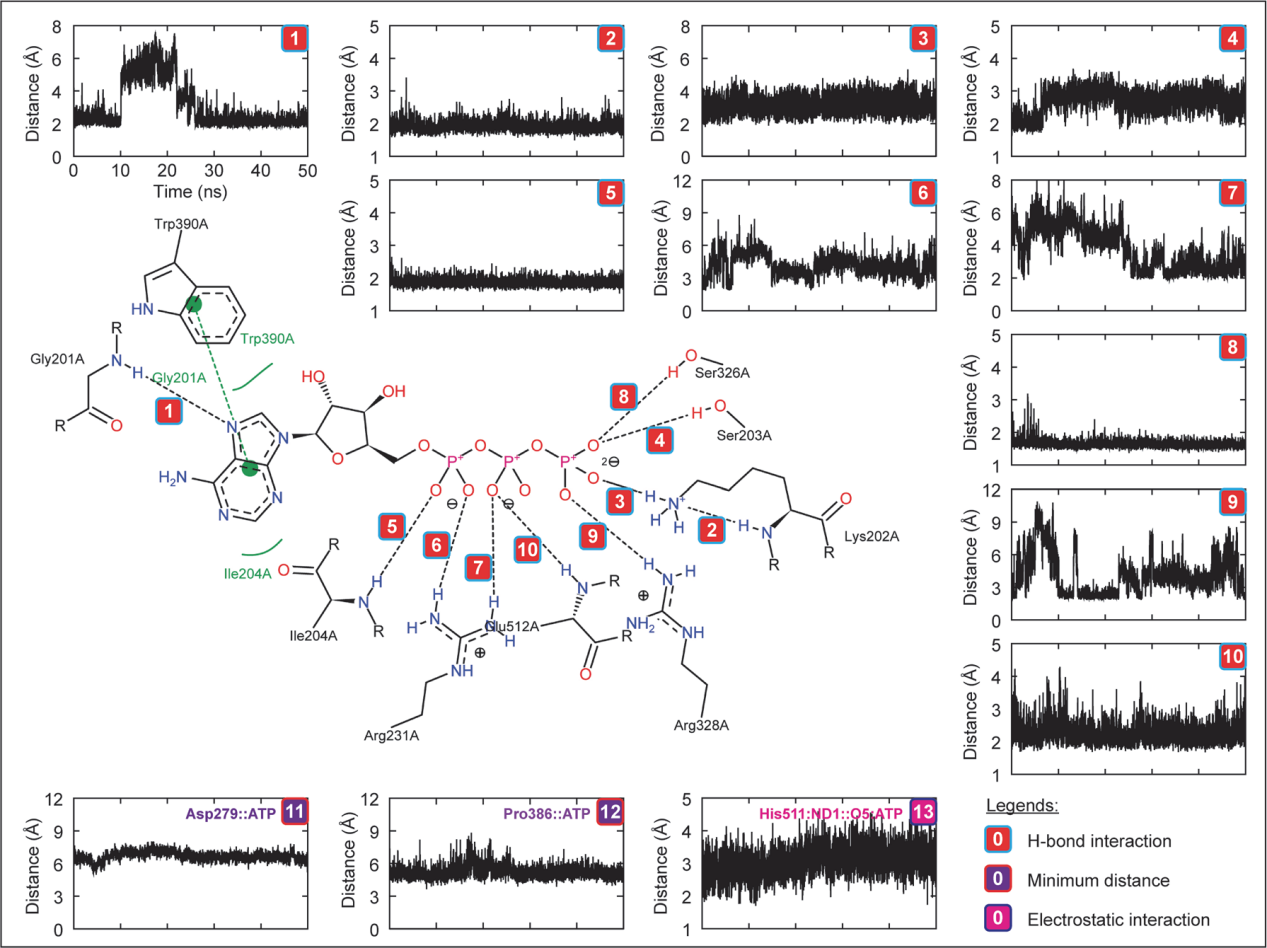
The figure shows the intermolecular interactions and their distances formed between zNOD1-NACHT and ATP (Complex III) in the final representative structure obtained after of 50 ns MD simulation. The figure accompanies the distance of each observed H-bonds, specified electrostatic interaction and hydrophobic contacts. The colors in boxes indicate different interactions; red indicates H-bond interaction, violet designates electrostatic interaction and magenta represents minimum distance between ATP and residue.

**Fig 8:**
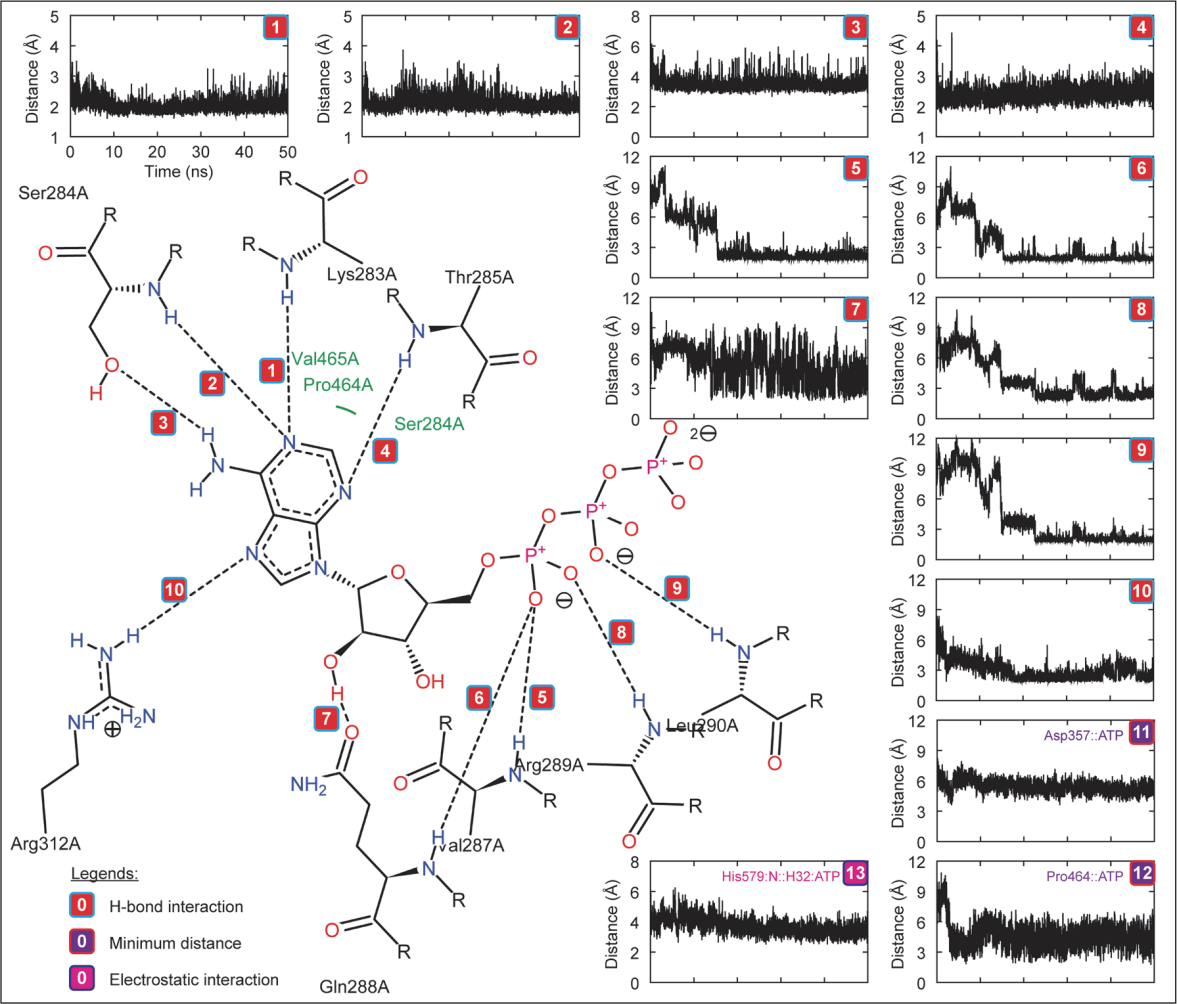
Intermolecular interaction and distance analysis of ATP with the final representative structure of zNOD2-NACHT obtained after of 50 ns MD simulation (Complex I). The figure displays the distance of each observed H-bonds, specific electrostatic interaction and minimum distances. The colors in boxes indicate different interactions; red indicates H-bond interaction, blue designates electrostatic interaction, and magenta represents minimum distance between ATP and residue.

To understand the molecular interaction of ATP with zNOD1, zNOD2 and mNLRC4-NACHT domain at atomic level, we analyzed the H-bonds formed between receptor and ligand during MD simulation. The final snapshots of complexes from MD trajectories were taken as the reference structures for interaction analysis. DS visualizer and PoseView were used for interaction mapping, visualization and exploration. Furthermore, the distance of H-bonds formed between NACHT domains and ATP was calculated using *g_dist* program in GROMACS. In NOD1, Gly201, Lys202, Ser203, Ile204, Arg231, Arg328, Ser326 and Glu512 formed 10 discrete H-bonds with ATP with an average interatomic distance of ~2.1 Å. Moreover, Met205, Pro386, Leu387, Trp390 and Leu510 showed strong hydrophobic contacts, whereas Asp279, His511 and Thr513 were involved in electrostatic interaction with ATP (Fig. 7, Table 5A). In contrast, zNOD2-NACHT-ATP complex revealed 10 intermolecular H-bonds involving residues Lys283, Ser284, Thr285, Val287, Gln288, Arg289, Leu290 and Arg312. Leu286, Val465, Pro464 and Trp468 formed hydrophobic contacts with ATP and Asp357 and His579 formed electrostatic interactions (Fig. 8, Table 5B), thereby creating support for the complex to hold ATP in the active site pocket of zNOD2. In case of zNOD2, the average H-bond distance was found to be ~2.3 Å. In mNLRC4, Gly172, Lys173, Gly174, Lys175, Ser176, Thr177 and Arg181 formed strong with H-bonds (average interatomic distance ~2.1 Å) with ATP. Further, Leu178, Pro338, Leu339 formed hydrophobic contacts and electrostatic interaction was governed by Glu170, Ser171, His204, Asp248 and Thr278 residues. But, His443 (of NLRC4 Sensor 2) did not form strong electrostatic interaction as that of conserved ‘His’ in NOD1 and NOD2.

**Table 5:** Molecular interaction of ATP with zNOD1 (A), zNOD2 (B) and mNLRC4-NACHT after molecular dynamics simulation. The critical amino acids involved in ATP binding are highlighted in bold font.

**(A) NOD1-NACHT-ATP**					
**Hydrogen Bond**			**Hydrophobic Interaction**	**Electrostatic Interaction**	**Van der Walls Interaction**
**NACHT**	**ATP**	**length**			
**Gly201:HN**	N2	2.0	Met205,	**Asp279,**	Gly199,
**Lys202:HN**	**O2**	**2.0**	**Pro386,**	**His511,**	Val200,
**Lys202:HZ3**	**O1**	**2.3**	Leu387,	Thr513	Lys208,
**Ser203:HN**	O2	2.4	Trp390,		Gly280,
**Ile204:HN**	O7	1.7	Leu510		Tyr281,
**Arg231:HH12**	O4	2.0			Lys345,
**Arg231:HH22**	O8	2.0			Lys394,
**Ser326:HG**	O3	2.7			Glu508
**Arg328:HH21**	**O1**	**2.0**			
**Glu512:HN**	O4	2.3			
**(B) NOD2-NACHT-ATP**					
**Lys283:HN**	**N4**	**2.2**	Leu286,	**Asp357,**	Ala279,
**Ser284:HN**	N4	2.1	Val465,	**His579**	Gly282,
**Ser284:OG**	N3	3.2	**Pro464,**		His291,
**Thr285:HN**	N5	2.1	Trp468		Ser310,
**Val287:HN**	O8	1.8			Lys423,
**Gln288:HN**	O8	2.2			Phe425,
**Gln288:OE1**	H1	3.4			Phe433,
**Arg289:HN**	O4	1.9			Leu578
**Leu290:HN**	O4	2.0			
**Arg312:HH22**	N2	2.1			
**(C) mNLRC4-NACHT-ATP**					
**Gly172:H**	O1	1.7	Leu178	Glu170	Ile182
**Lys173:H**	O2	1.8	**Pro338**	Ser171	Met300
**Gly174:H**	O2	1.6	Leu339	His204	Leu308
**Lys175:HZ3**	**O1**	**2.7**		**Asp248**	Lys440
**Lys175:H**	**O2**	**2.2**		Thr278	
**Lys175:H**	**O4**	**2.3**			
**Ser176:H**	O3	1.7			
**Ser176:HG**	O3	2.1			
**Thr177:HG**	O4	2.8			
**Thr177:H**	O4	2.1			
**Arg181:HH11**	N4	2.5			
**Arg181:NH**	N4	2.2			

**Fig 9:**
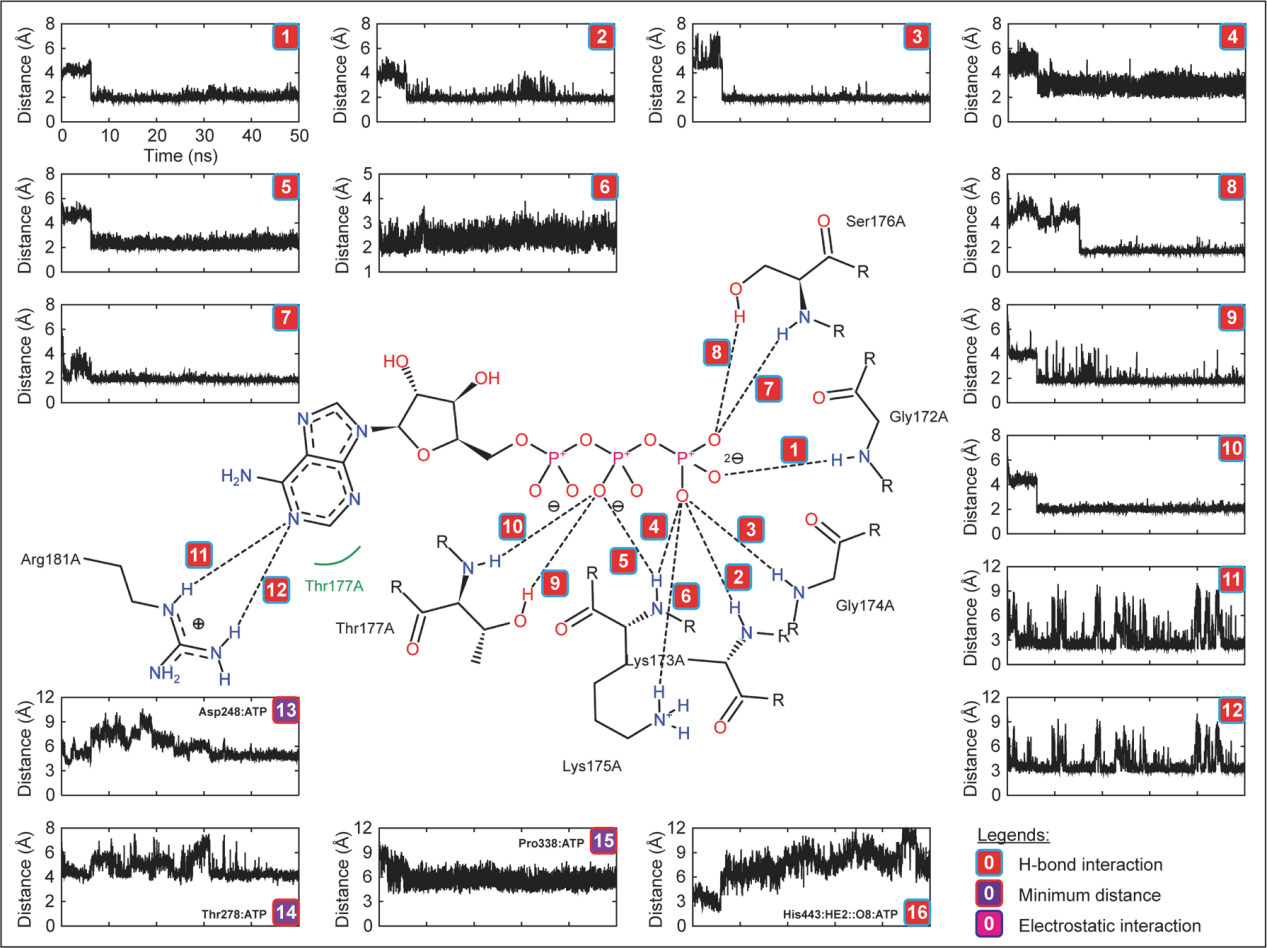
The figure indicates intermolecular interaction and distance analysis of ATP with the final representative structure of mNLRC4-NACHT obtained after 50 ns MD simulation. The distance of each observed H-bonds, specific electrostatic interaction and minimum contact between ATP and residue. The colors in boxes indicate different interactions where red indicates H-bond interaction, violet designates electrostatic interaction, and magenta represents close contacts.

In a recent mutagenesis study, Zurek *et al*. have revealed that Lys208, Asp284, Arg333, Pro391 and His517 of hNOD1, and Lys305, Asp379 and Pro486 of hNOD2 were critical for ATPase activity [19]. Consistent with hNOD-NACHT domains, our study demonstrated that in zNOD1, Lys202 of Walker A motif (Lys208; hNOD1) formed two strong H-bonds with γ-phosphate group of ATP, however, in zNOD2; Lys283 formed one H-bond with adenine moiety of ATP. The first acidic residue of Walker B (Asp279; zNOD1 and Asp357; zNOD2) formed weak electrostatic interaction with ATP with an average distance of ~6 Å. Earlier studies have shown that first acidic residue of Walker B is responsible for Mg^2+^ coordination [19,63]. Hence, our findings perfectly corroborate with that of the experimental evidence of human counterpart signifying the conserved role of Aspartic acid. Arg328 of zNOD1 Sensor 1 formed a strong H-bond with γ-phosphate group of ATP, whereas the conserved Arg404 was not involved in interaction. Pro386 of zNOD1 and Pro464 of zNOD2 GxP motif showed mild hydrophobic contacts with adenine moiety, reflecting a clear synchronization with human counterpart [19]. His511 of zNOD1 Sensor 2 formed a strong electrostatic interaction with γ-phosphate group and His579 of zNOD2 interacted electrostatically with ‘adenine moiety’ of ATP. As compared to recognition of ATP by zNOD1 and zNOD2, we found notable changes in mNLRC4. Likewise, zNOD1 and zNOD2 ‘Lys175’ (Walker A) formed three strong H-bonds, Asp248 interacts electrostatically (average distance of ~4.5Å) with γ-phosphate group of ATP and Pro338 showed weak hydrophobic contact with adenine moiety. In contrast, we came across no such interaction of ‘Thr279’ (of mNLRC4 Sensor 1) exist, however the adjacent ‘Thr278’ formed strong electrostatic interaction with γ-phosphate group (Fig. 9; Table 5C). Earlier studies by Proell *et al*. and Hanson *et al*. have revealed that ‘His’ of Sensor 2 (WHD) is responsible for nucleotide binding, hydrolysis, and coordination of γ-phosphate group [13,64]. In addition, Zurek and coworkers have reported that a single point mutation (His->Ala on ATPase activity) in NOD1 showed complete loss of NF-κB signaling. In contrast the equivalent mutation in NOD2 showed reduced NF-κB activation [19]. In mNLRC4, the mutation of His443->Leu443 resulted lower expression of interleukin (IL)-1β than wild-type mNLRC4 in response to ATP [28]. As evident from distance calculation, we found that His443 interacts electrostatically with γ-phosphate group for first 5 ns which goes on an increasing trend thereafter till 50 ns (Fig. 9) which supports the earlier studies by Hu *et al*. [28]. Our results shows that His511 and His579 of Sensor 2 showed strong electrostatic/H-bond interaction with ATP in both NOD1 and NOD2 (Fig. 7, 8). Due to the differential binding mode of ATP, His579 is not able to coordinate with γ-phosphate group, which is thus not anticipated to participate in hydrolysis; a major process of ATPase activity [8,19]. Thus, it may be hypothesized that the conserved ‘His’ (of Sensor 2/WHD) plays pivotal and differential role in ATPase activity of different NLRs.

**Fig 10:**
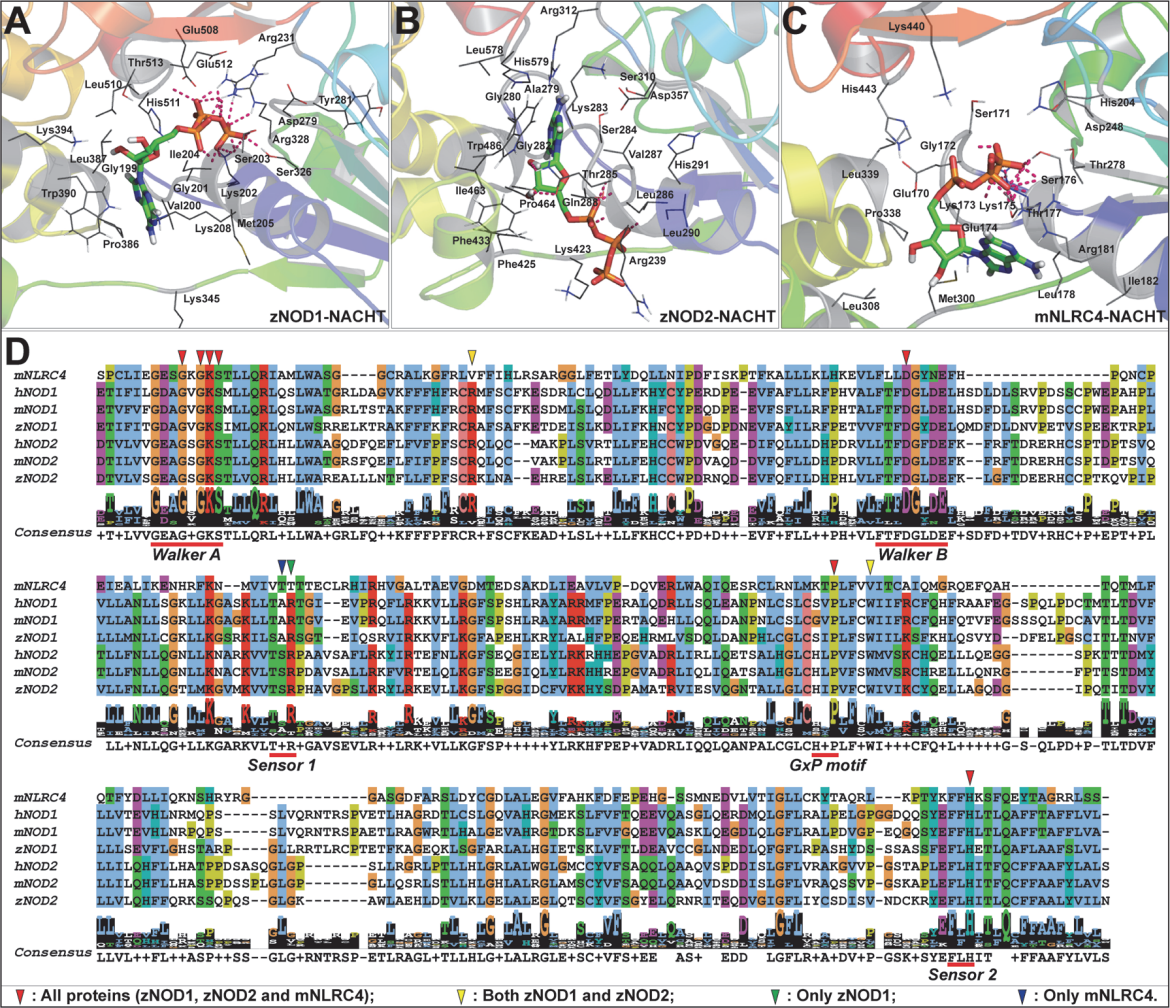
ATP binding poses in zNOD-NACHT domains in simulated models zNOD1-NACHT-ATP complex (A), zNOD2-NACHT-ATP complex (B) and mNLRC4-NACHT-ATP complex (C). The protein is shown as cartoon; interacting amino acids are shown as lines and ATP as stick.9D) Multiple sequence alignment of NACHT domains of NOD1 and NOD2 (from human, mouse and zebrafish)with mouse NLRC4 sequences. The key functional motifs are underlined and labeled. The potential ATP binding residues are pointed by different colored triangles.

A detailed interaction of zNOD1, zNOD2 and mNLRC4-NACHT with ATP is presented in Fig. 10A-C. The atomic scale illustration clearly shows the differential binding mode of ATP in NOD1 and NOD2. So as to confirm the residues involved in ATP recognition, we performed a sequence alignment of human, mouse and zebrafish NACHT domains (of NOD1 and NOD2) with mNLRC4-NACHT to locate the conserved residues responsible for ATP interaction (Fig. 10D). Arg231 of zNOD1 and Arg312 of zNOD2 were found to be conserved and formed strong H-bonds with ATP. In contrast to NOD1 and NOD2, ‘Arg’ was replaced by ‘Val’ in mNLRC4 and didn’t form any interaction with ATP. Our study also highlighted that the conserved ‘Trp’ (Trp390 of zNOD1 and Trp486 of zNOD2) play a vital role in maintaining strong hydrophobic contact with ATP (Table 5), however in mNLRC4, ‘Val’ was replaced by ‘Trp’ and no interaction was seen during the course of simulation.

### I. Essential dynamics analysis

**Fig 11:**
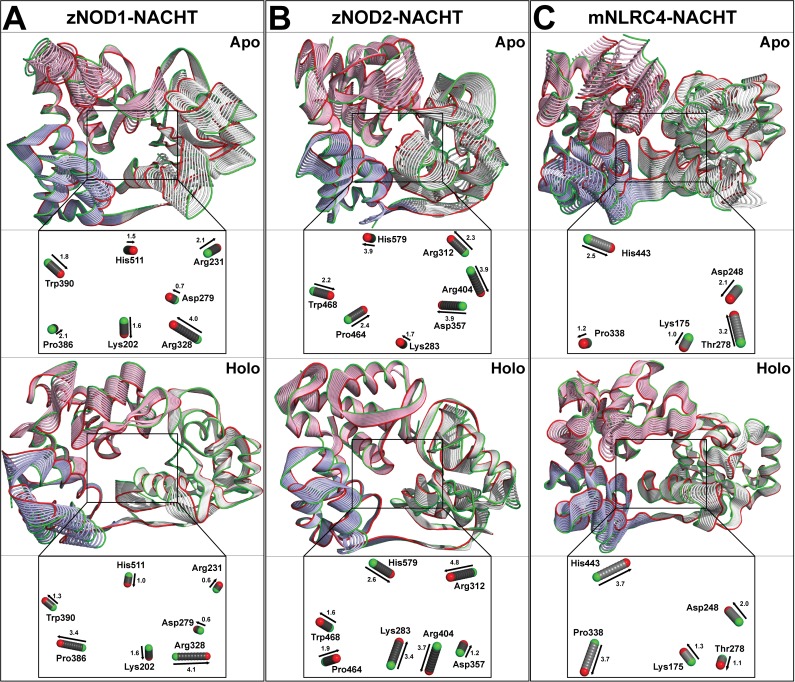
Essential dynamics analysis of apo and holo conformers. Global motion of zNOD1-NACHT (A), zNOD2-NACHT (B) and mNLRC4-NACHT (C). The principal component analysis was performed using g_covar and g_anaeig program of GROMACS and protein motions are visualized in PyMOL. The apo and holo state conformations are labeled, and the magnitude and direction of displacement of the key ATP binding residues are labeled and shown below the respective 3D-conformers. Green and Red colors represent initial and final conformation respectively. The protein is represented in the tube model and Cα atoms are represented in sphere.

The activity of NOD-NACHT domain was correlated with the protein motion in both apo and holo conformations through essential dynamics calculation. Ligand binding greatly influences the overall protein motion, and such functional internal motions regulate the protein functions. Structural rearrangements are crucial for the initiation of oligomerization and signaling. The self-oligomerization mediated upon ATP binding in NOD-NACHT indicated a conformational alternation required for NOD-signaling [8,19]. A comparative analysis of the overall motion in both apo and holo forms distinctly portrayed that the apo state (zNOD1, zNOD2 and mNLRC4) NACHT-conformers were highly flexible depicting a substantial motion as compared to holo forms. The internal motions of NACHT domains in both apo and holo states were presented in Fig. 11.

A close inspection of the NOD1/2/NLRC4-NACHT domains revealed that the motion at ATP binding cleft were restricted in the apo form. On the other hand, in holo state the α-helices and β-sheets adjacent to ATP binding pocket (*i*.*e*., Walker A, Walker B, Sensor I, GxP motif and Sensor 2) exhibited a significant motion. Furthermore, calculation of the principal movement of Cα atoms from each critical residue involved in ATP binding magnified the significant motile regions at the active sites. The Lys202 (Walker A; involved in nucleotide binding) showed inward movement with a displacement of 1.6 Å in both apo and holo systems of NOD1 (Fig. 11A). Residue Asp279 (Walker B) presented a little displacement in both states, whereas Arg328 (Sensor 1) showed differential inward and outward movement with a displacement of 4.1 and 4.0 Å, respectively. Pro386 (GxP motif) displayed inward movement (2.1 Å) in apo and outward movement (3.4 Å) in holo system. Another key active site residue Trp390 showed both inward (1.8 Å) and outward movements (1.3 Å) in apo and holo conformers, respectively. His511 of Sensor 2 exhibited differential displacement for both the systems. In NOD2, Lys283 (Walker A) of NOD2 in apo condition showed upward movement (1.7 Å) whereas in holo condition, it displayed inward movement (3.4 Å) (Fig. 11B). Asp357 (Sensor 1) exhibited inward (3.9 Å) and outward (1.2 Å) motions in apo and holo state, respectively. Arg404 (Sensor 1) showed a large outward movement in both apo and holo conditions. Pro464 (of GxP motif), Trp468, and His579 (of Sensor 2) showed differential displacements for both conformers. As compared to zNODs, the Walker A and B residue (Lys175 and Asp248 of mNLRC4) showed contrasting movements in apo and holo form (Fig. 11C). The significant movement was noticed in ‘Pro338’, in apo form the movement is upward up to 1.2 Å, whereas in holo form the movement is inward (3.7 Å). The differential movement of His443 was recorded with divergent displacements. Overall, these findings indicated a comparatively stable holo conformation. The residual motions associated with ATP binding pockets could be used to understand the structural alternations and signal transformation in NOD receptors.

Both NOD1 and NOD2 are intracellular PRRs of the NLR gene family responsible for innate immune responses. NACHT domain located centrally in both NOD1/2 displays ATPase activity, which is indispensable for activation and oligomerization leading to wide array of inflammatory signaling responses [7–10,19]. Several studies have shown that NOD1/2 along-with other members of NLR family (*i*.*e*., NLRP1, NLRP3, NLRP12 and NLRC4 etc.) prefer to bind and hydrolyze ATP over other nucleotides [28, 65–67]. A recent study by Zurek *et al*., have shown that NOD1 and NOD2 explicitly employ different modes of activation. Interestingly, in their study they have reported that mutation in conserved ‘His’ (Sensor 2 of WHD) in the NACHT domain showed contrasting effects on NOD1 and NOD2 mediated NF-κB activation. In our study, the minute observation have clearly depicted differential mode of ATP binding; where, γ-phosphate group of ATP protrudes towards the central cavity in zNOD1. In contrast, adenine moiety protrudes towards the central cavity (in zNOD2), as a result Arg328 (zNOD1) interacts with γ-phosphate group of ATP whereas Arg404 of zNOD2 didn’t form any contact with ATP. Conserved ‘His’ (as mentioned above) has a crucial role in NLR activation. In zNOD1, His501 forms an H-bond/strong electrostatic interaction with γ-phosphate group; however a weak electrostatic interaction was noticed in His579 (zNOD2). In contest to zNOD1 and zNOD2, mNLRC4 showed differential interaction during the course of MD simulation (as discussed above). This, in turn, raises an important question that whether the functional role of conserved ‘His’ in NLRC sub-family diverged. Zurek *et al*., suggested mutation Glu382->Lys382 (extended Walker B of NOD2) showed high level of NF-κB activation in the absence of MDP stimulation, but in NOD1 the conserved Glu288 (upon mutation) did not show any activation of NF-κB signaling [19]. But, from our computational study, we couldn’t establish the role of extended Walker B acidic residues of zNOD1 and zNOD2 (Glu283 and Glu361), which require further mutational studies to explain their role in NF-κB signaling.

NLRs are generally activated by pathogenic ligands by LRRs and subsequently nucleotides bind to NACHT domain follows oligomerization and interaction of adaptor molecule *via* CARD-CARD interaction thereby aid in down-stream signaling for NF-κB activation [7–10]. But current literature states that NOD2 can rapidly cycle between the ATP-bound and open state while signaling is inactive. Binding of ATP in NOD2-NACHT induces a conformational change which is dependent on intact Walker A and Walker B motifs that leads to further ligand (MDP) recognition and assembly of a signaling-competent complex. Our data supports the NOD2 interactions with ATP and induces a change in the conformation as compared to zNOD1 and mNLRC4 global movement during the course of MD simulation.

Apart from playing an essential role in nucleotide binding and oligomerization in NOD signaling pathway, it also plays an important role in interaction with adopter protein molecule CARD9 [68] which is crucial in NOD2-mediated p38 and JNK signaling pathway. NOD2 possess two discrete binding sites *i*.*e*., NACHT or linker joining adjacent NACHT and CARD domain responsible of NF-κB signaling. Due to absence of high resolution structural homologue of linker joining adjacent domains *viz*. CARD-NACHT-LRR in both NOD1 and NOD2, it is quite difficult to establish inter-domain functional relationship in response to PAMPs, nucleotides and adaptor molecules. Further, as the whole study is based on computational approach, one should not overlook the limitations and pitfalls of these approaches in rationalizing the key defense signaling pathway. Even through computational methods have been widely used to obtain atomistic insights into molecular recognition process, it is quite difficult to establish the genuine mechanism without high resolution experimental X-ray crystallography and NMR spectroscopy techniques. Furthermore, computational techniques rely exclusively on the precision of inherent scoring functions, algorithms, and availability of high computational cost. The advantages and limitations of these protocols have been well discussed in recent reports [69–72].

## Conclusion

Here we reported hypothetical ATP binding mechanism of zNOD1 and zNOD2-NACHT domains using comparative modeling, molecular docking and MD simulation techniques. The findings of our study revealed a differential binding mode of ATP in the catalytic cavity of zNOD1 and zNOD2-NACHT domain which was well supplemented with mNLRC4-ATP interaction. In zNOD1-NACHT, γ-phosphate group of ATP was deeply buried inside the central cavity as in mNLRC4, whereas adenine moiety positioned outward from the core cavity. On the other hand, ATP in zNOD2-NACHT showed a reverse binding mode with adenine moiety protruding toward and γ-phosphate group away from the core cavity. Essential dynamics analysis suggested that the NACHT domains were highly rigid in holo conformation as compared to the apo form. In addition, our predictions highlight the role of critical amino acids responsible for ATP binding in NLRC sub-family members. Further studies are required to understand the functional relevance of inter-domain behavior in response to recognition of PAMPs, nucleotide, and adaptor molecules. Altogether, this study hypothesized the novel insights into ATP binding mechanism in zebrafish NOD-NACHT domain which could be used to understand the NOD-mediated innate immune signaling transduction in other lower and higher eukaryotes.

## Supporting Information

S1 Fig Multiple sequence alignment of human, mouse, and zebrafish NOD1 (A) and NOD2 (B) sequences shows the domain margins. The three domain architecture viz. CARD/s, NACHT [NBD-HD1-WHD], HD2 and LRRs is shown in different colored boxes; brown (CARD/s), green (NBD), orange (HD1), gray (WHD), purple blue (HD2) and red (LRR). The symbols ‘*’, ‘:’ and ‘.’, represents identical, conserved and semi-conserved substitutions of amino acids respectively.
(TIF)

S2 Fig Discrete optimized protein energy (DOPE) score profile of the zNOD1and zNOD2-NACHT models. The DOPE score profiles of I-TASSER models were chosen for structural analysis.
(TIF)

S3 Fig Secondary structure prediction of zNOD1 and zNOD2-NACHT domains in PSIPRED server. The secondary structural elements are shown inside the legend box. The domain boundaries are presented in different colored lines and presented in legend boxes.
(TIF)

S4 Fig Validation reports of zNOD1 and zNOD2-NACHT models by Ramachandran plot, ProSA and ERRAT. (TIF)

S5 Fig Comparative analysis displaying root mean fluctuations (RMSF) of apo and holo systems of zNOD1-NACHT (A), zNOD2-NACHT (B) and mNLRC4-NACHT (C). Different colored lines indicate different simulation systems.
(TIF)

S6 Fig Secondary structures of (A) zNOD1-NACHT and (B) zNOD2-NACHT as a function of simulation time. Magenta, yellow, blue and white segments indicate α-helix, β-sheet, turn and coil, respectively.
(TIF)

S1 Table Atomic compositions and properties of different simulation systems. (PDF)
