# Supplementary figures and images for: Structural Models of Zebrafish (Danio rerio) NOD1 and NOD2 NACHT Domains Suggest Differential ATP Binding Orientations: Insights from Computational Modeling, Docking and Molecular Dynamics Simulations
Source: PLoS One. 2015 Mar 26;10(3):e0121415. doi: 10.1371/journal.pone.0121415 (PMC4374677; doi:10.1371/journal.pone.0121415)

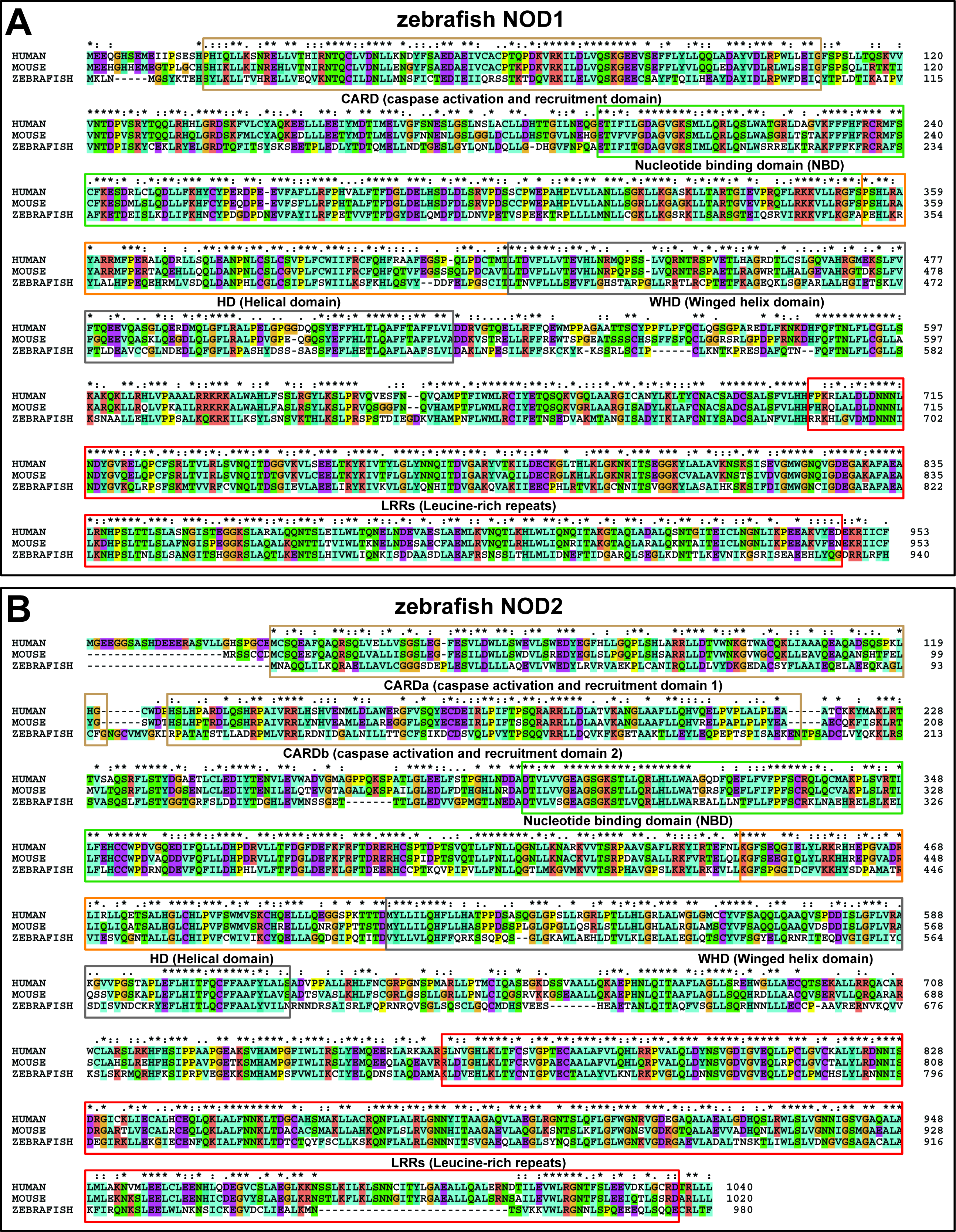

Supplement: S1 Fig — The three domain architecture viz. CARD/s, NACHT [NBD-HD1-WHD], HD2 and LRRs is shown in different colored boxes; brown (CARD/s), green (NBD), orange (HD1), gray (WHD), purple blue (HD2) and red (LRR). The symbols ‘*’, ‘:’ and ‘.’, represents identical, conserved and semi-conserved substitutions of amino acids respectively. (TIF) [file pone.0121415.s001.tif]

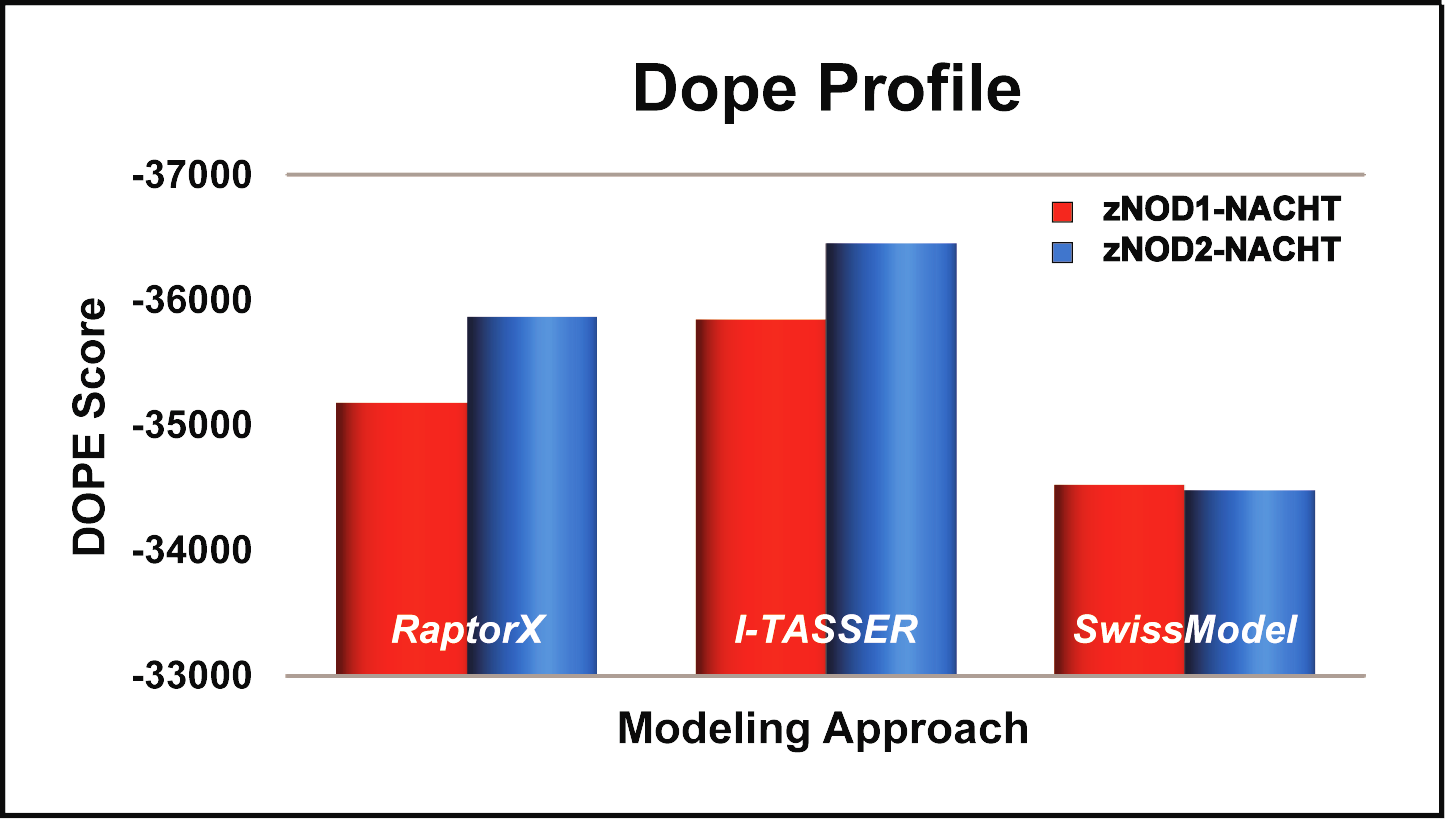

Supplement: S2 Fig — The DOPE score profiles of I-TASSER models were chosen for structural analysis. (TIF) [file pone.0121415.s002.tif]

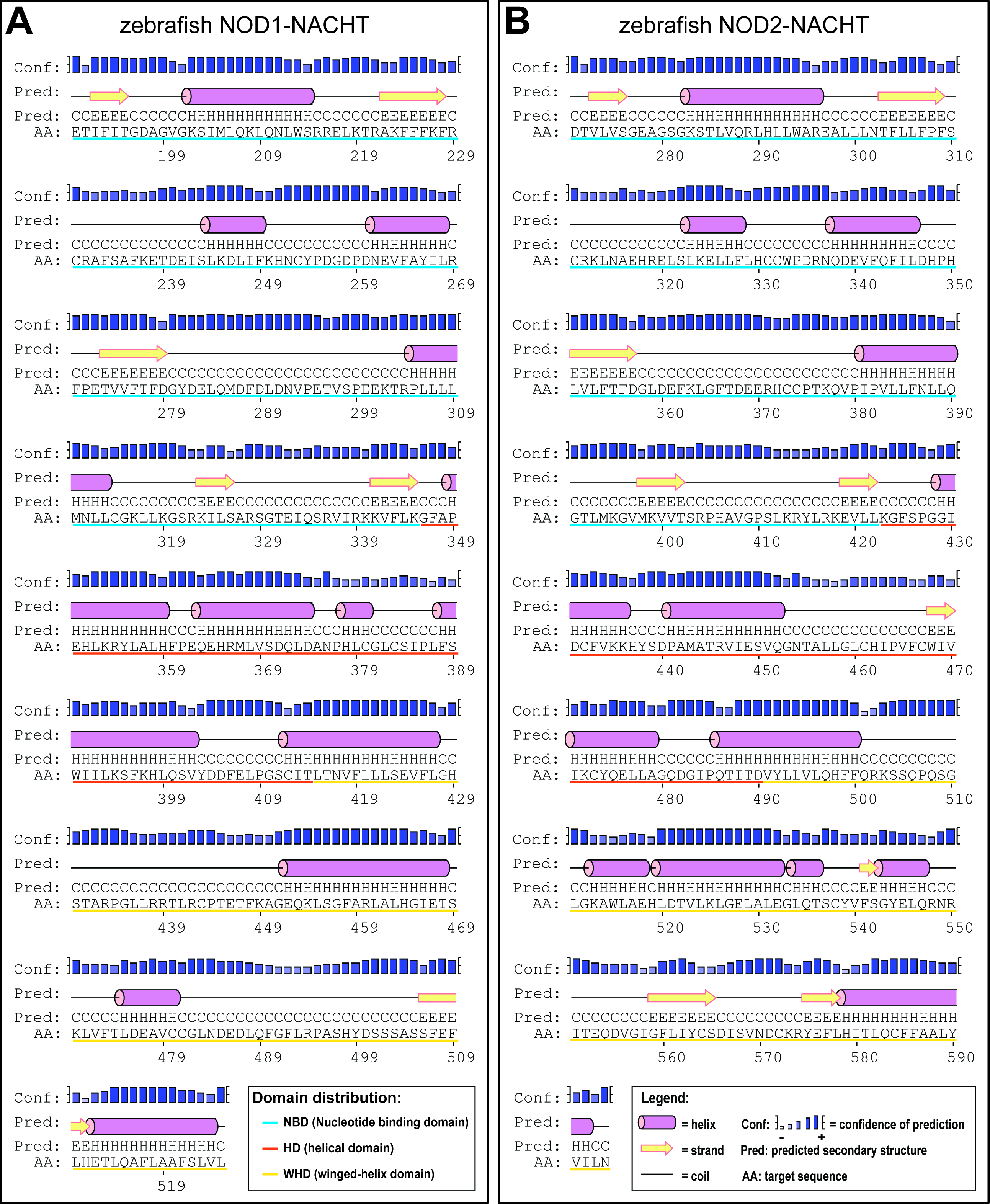

Supplement: S3 Fig — The secondary structural elements are shown inside the legend box. The domain boundaries are presented in different colored lines and presented in legend boxes. (TIF) [file pone.0121415.s003.tif]

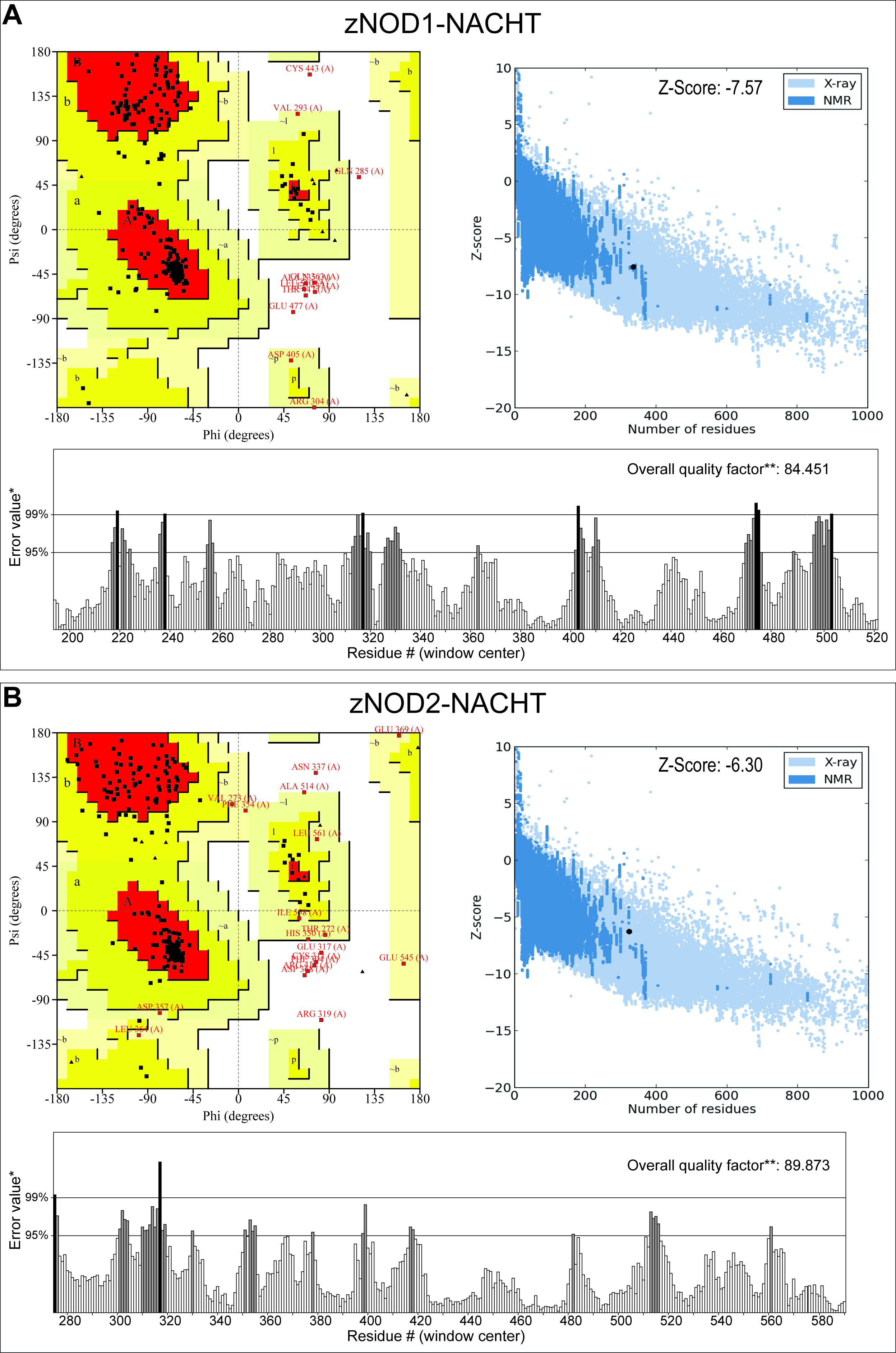

Supplement: S4 Fig — (TIF) [file pone.0121415.s004.tif]

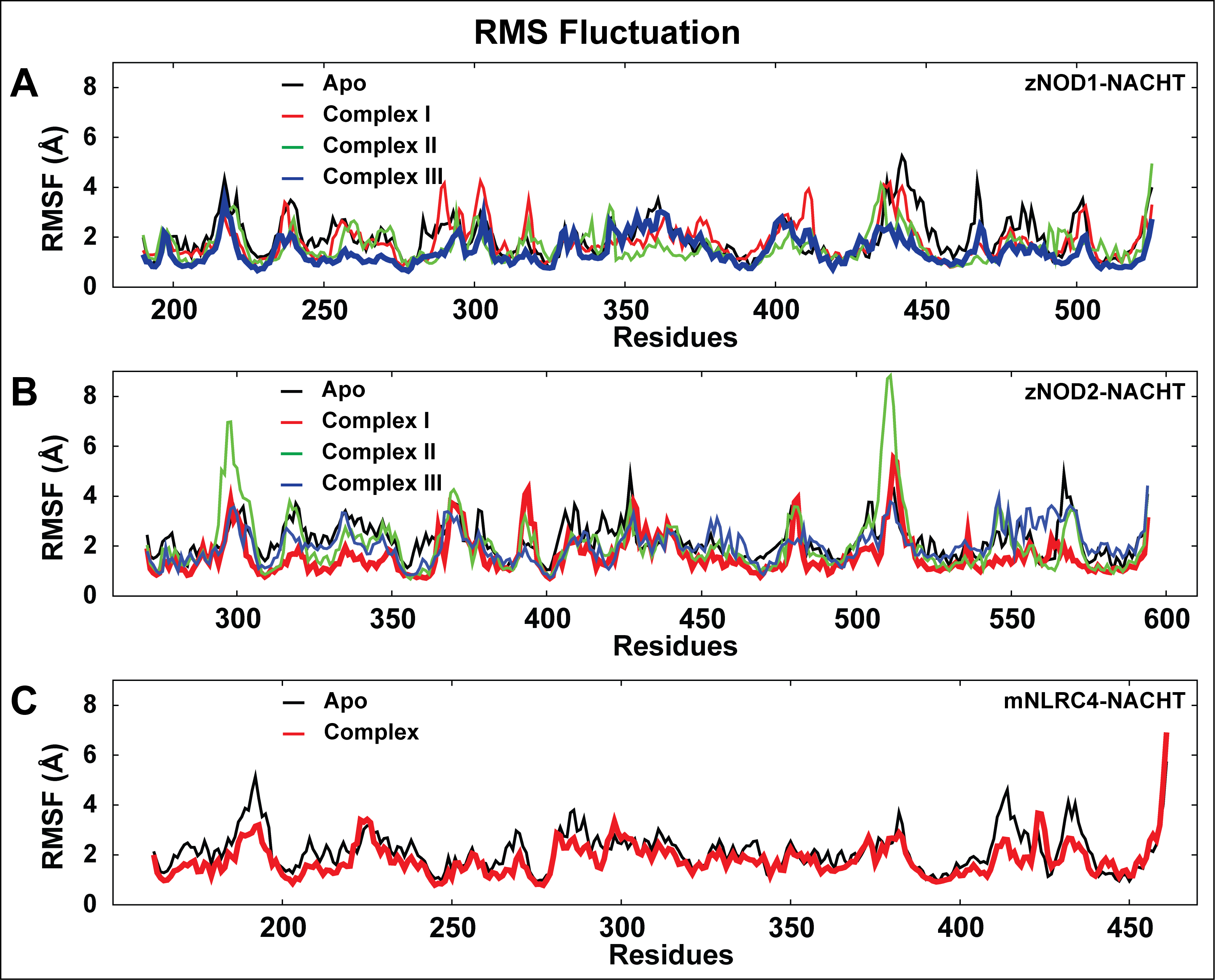

Supplement: S5 Fig — Different colored lines indicate different simulation systems. (TIF) [file pone.0121415.s005.tif]

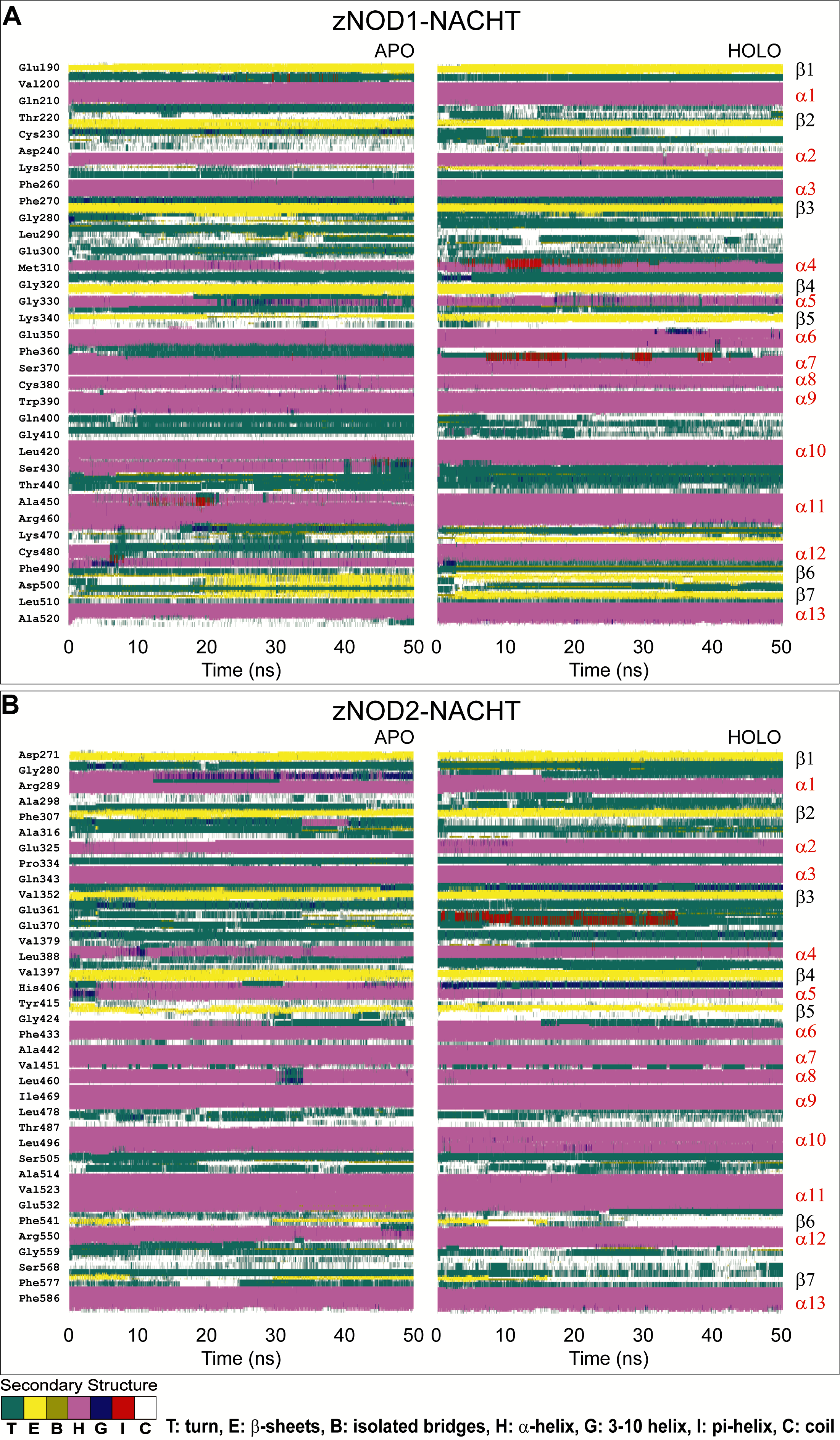

Supplement: S6 Fig — Magenta, yellow, blue and white segments indicate α-helix, β-sheet, turn and coil, respectively. (TIF) [file pone.0121415.s006.tif]
