# Supplementary material for: Structural Models of Zebrafish (Danio rerio) NOD1 and NOD2 NACHT Domains Suggest Differential ATP Binding Orientations: Insights from Computational Modeling, Docking and Molecular Dynamics Simulations
Source: PLoS One. 2015 Mar 26;10(3):e0121415. doi: 10.1371/journal.pone.0121415 (PMC4374677; doi:10.1371/journal.pone.0121415)
Supplement: S1 Table — (PDF) [file pone.0121415.s007.pdf]

Table S1

Atomic compositions and properties of different simulation systems

Simulation system	Number of water molecule	Number of Na^+	Number of Cl^-	Total number of atoms
**Apo System**				
zNOD1-NACHT (336 aa)	20884	64	62	66251
zNOD2-NACHT (324 aa)	19944	61	59	63259
**zNOD1-NACHT (336 aa) + ATP (Holo system)**				
Complex I	20862	68	62	66226
Complex II	20859	68	62	66217
Complex III	20883	68	62	66289
**zNOD2-NACHT (324 aa) + ATP (Holo system)**				
Complex I	19923	65	59	63237
Complex II	19920	65	59	63228
Complex III	19956	65	59	63336
